# GDF15 Ameliorates Deoxynivalenol‐Induced Anemia by Resolving Ribosomal Stress–Mediated Erythropoietic Arrest

**DOI:** 10.1002/advs.202509265

**Published:** 2025-09-03

**Authors:** Yan Li, Peijun Jia, Jingxin Zhang, Huan Zhang, Bing Li, Jiawei Chang, Yating Li, Longzhen Zhao, Yazhe Zhen, Tingting Zheng, Yuanlin Xu, Xiuli An, Shijie Zhang

**Affiliations:** ^1^ School of Life Sciences Zhengzhou University Zhengzhou Henan 450000 China; ^2^ Department of Internal Medicine The Affiliated Cancer Hospital of Zhengzhou University & Henan Cancer Hospital Zhengzhou Henan 450008 China; ^3^ Laboratory of Membrane Biology New York Blood Center New York NY 10065 USA

**Keywords:** anemia, cell cycle arrest, DON, erythropoiesis, GDF15

## Abstract

Deoxynivalenol (DON) is a prevalent trichothecene mycotoxin that contaminates global food supplies, posing significant health risks; however, targeted therapies against DON are scarce. Although DON‐induced anemia is well‐documented, the underlying mechanisms remain unclear. In this study, the effects of DON on erythropoiesis are examined in detail using complementary murine models and human primary erythroid cultures. DON impaired erythropoiesis by disrupting hematopoietic homeostasis as well as erythroid commitment and differentiation via ribosomal stress–mediated cell cycle arrest. Multi‐omics analyses revealed that the effects of DON are attributed to ribosomal dysfunction, which selectively disrupted protein synthesis without altering mRNA expression. Mechanistically, DON downregulated translation of growth differentiation factor 15 (GDF15) along with decreases in β‐catenin, Myc, and p21. Importantly, GDF15 supplementation rescued DON‐induced erythropoietic defects in vitro and in vivo, restored levels of β‐catenin, Myc, and p21, and cell cycle progression, indicating DON inhibits erythropoiesis via the GDF15–β‐catenin–Myc–p21 axis. These findings elucidate the pathogenesis of DON‐induced anemia and identify GDF15 as a novel therapeutic target against mycotoxin poisoning.

## Graphical abstract

Deoxynivalenol induces anemia by disrupting hematopoietic homeostasis and impairing erythroid lineage commitment and differentiation via ribosomal stress–mediated G1/S cell cycle arrest. GDF15 supplementation alleviates this arrest through the β‐catenin–Myc–p21 signaling axis, highlighting GDF15 as a potential therapeutic target for DON‐induced hematotoxicity.

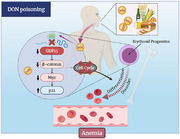

## 1 Introduction

Deoxynivalenol (DON), a trichothecene mycotoxin colloquially referred to as “vomitoxin,”^[^ ^1^, ^2^, ^3^ ^]^ exhibits remarkable chemical stability throughout crop growth, harvest, and processing owing to its distinctive molecular structure.^[^ ^4^, ^5^ ^]^ Alarmingly, DON contamination is pervasive in global grain supplies, having significant public health implications. A European surveillance study analyzing over 40000 food samples detected DON contamination in ≈ 57% of specimens.^[^ ^6^ ^]^ Similarly, monitoring programs in the United States have identified DON in ≈ 73% of wheat and 92% of corn samples.^[^ ^6^ ^]^ In China, the largest gastroenteritis outbreaks during 1984–1991 that affected 130000 individuals were linked to the consumption of cereals contaminated with DON and/or other trichothecenes.^[^ ^7^ ^]^ Therefore, DON poses a critical threat to food safety and public health.

At the cellular level, DON affects cell proliferation, differentiation, cell signaling, and macromolecular synthesis, thereby impairing gastrointestinal homeostasis, neuroendocrine function, and immune responses.^[^ ^8^, ^9^ ^]^ Its pro‐apoptotic effects are well‐documented in both cancerous and normal cells within the digestive and immune systems.^[^ ^8^, ^10^, ^11^, ^12^ ^]^ Moreover, DON binds to the peptidyl transferase active site of ribosomes, impeding peptide bond formation and ribosomal function.^[^ ^13^ ^]^ It also affects precursor messenger RNA splicing and perturbs protein synthesis.^[^ ^14^ ^]^ However, clinically effective therapeutic interventions against DON toxicity are currently lacking.

In addition to its effects on digestion and immunity, DON has been shown to dysregulate the hematopoietic system due to its rapid absorption and distribution following exposure.^[^ ^15^ ^]^ Anemia is a common clinical manifestation of DON exposure.^[^ ^16^, ^17^ ^]^ Rainbow trout exposed to DON for 23 days exhibited reduced hemoglobin (HGB), mean corpuscular volume, and mean corpuscular hemoglobin.^[^ ^18^ ^]^ Pigs exposed to DON showed decreased red blood cell (RBC) and HGB contents.^[^ ^19^ ^]^ Similarly, in chicken models, varying degrees of RBC and HGB reduction associated with DON intake were observed, establishing red cell indices as sensitive biomarkers of DON toxicity.^[^ ^20^ ^]^ Elucidating the molecular mechanisms of DON‐induced anemia may facilitate therapeutic development.

Erythropoiesis originates from hematopoietic stem cells (HSCs) and progresses through multipotent progenitor cells (MPPs), common myeloid progenitor cells (CMPs), and megakaryocyte–erythroid progenitors (MEPs), followed by commitment to the erythroid lineage.^[^ ^21^, ^22^, ^23^ ^]^ Subsequently, erythroid progenitor cells, including burst‐forming unit‐erythroid (BFU‐E) and colony‐forming unit‐erythroid (CFU‐E) cells, undergo terminal erythroid differentiation to generate morphologically identifiable proerythroblasts, basophilic erythroblasts, polychromatic erythroblasts, and orthochromatic erythroblasts, which expel their nuclei to generate enucleated reticulocytes.^[^ ^24^, ^25^, ^26^ ^]^ As described above, although the correlation between decreased red cell indices and DON exposure was observed,^[^ ^18^, ^19^, ^20^ ^]^ whether and how DON affects erythropoiesis remains unclear. In this study, we employed murine models, human CD34^+^ cell cultures, and multi‐omics approaches to assess the erythropoietic toxicity of DON and define the underlying molecular mechanisms. We found interestingly that DON affected erythropoiesis via downregulation of growth differentiation factor 15 (GDF15). We further investigate the possibility of GDF15 supplementation as a novel therapeutic strategy.

## 2 Results

### 2.1 DON Induces Rapid Alterations in Peripheral RBC Indices and Disrupts Hematopoietic Homeostasis in Mice

**Figure 1:**
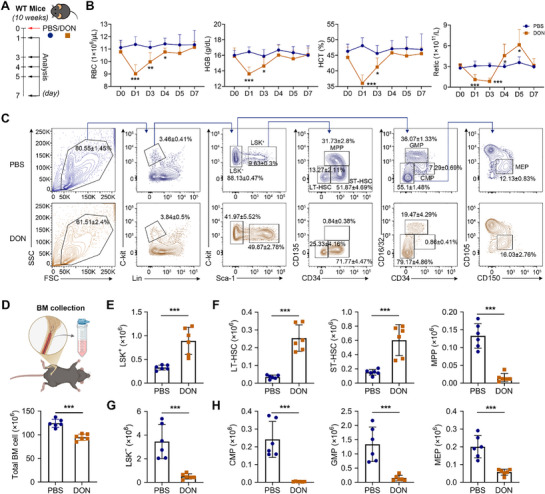
Deoxynivalenol (DON) causes maintenance and development imbalance in hematopoietic stem and progenitor cells (HSPCs). A). Schematic model of DON‐treated mice. B). Complete blood count of DON‐treated mice; *n* = 7 mice/group. C). Representative plots of flow cytometry analyses of HSPCs in the mice bone marrow (BM) on day 1 following treatment with phosphate buffer saline (PBS) or DON. D). Top: Schematic representation of the BM cell collection. Bottom: Quantification of total BM cells; *n* = 6 mice/group. The number of E). LSK^+^; F). LT‐HSC, ST‐HSC, and MPP; G). LSK^−^; and H). CMP, GMP, and MEP in the BM on day 1 following treatment with PBS or DON in mice; *n* = 6 mice/group. Data are presented as mean ± SD. Comparisons between two groups were performed using an unpaired two‐tailed Student's *t*‐test. Significance was set at ^*^p < 0.05, ^**^p < 0.01, and ^***^p < 0.001.

To investigate DON‐induced hematotoxicity, we selected a dose of 10 mg kg^−1^, which elicited significant hematological effects without mortality. Mice subjected to a single intraperitoneal DON injection (**Figure** **1**A) developed acute anemia, characterized by a rapid decline in peripheral RBC indices. RBC counts, HGB levels, and hematocrit (HCT) values decreased to their minimum levels on day 1 post‐exposure, followed by a gradual recovery between days 1 and 4 (Figure 1B). Concurrently, peripheral reticulocyte count, a sensitive indicator of bone marrow (BM) erythropoietic activity, was suppressed from days 1 to 3 (Figure 1B). These findings prompted us to examine the effects of DON on HSC homeostasis and progenitor cell differentiation. Compared with control mice, DON‐treated mice showed a significant reduction in total BM cells; however, an increase was observed in the absolute number of HSC‐enriched LSK^+^ cells (Lin^−^Sca‐1^+^c‐Kit^+^) (Figure 1C–E). Subpopulation analysis revealed elevated numbers of long‐ and short‐term HSCs in DON‐treated mice, accompanied by an increase in the S phase of the cell cycle process (Figure S1A,B, Supporting Information) and the proportion of Ki67^+^ cells (Figure S1C, Supporting Information), whereas MPPs were markedly depleted (Figure 1F). Further evaluation of the more differentiated LSK^−^ progenitor subset revealed significant reductions in the number of LSK^−^ cells, CMPs, MEPs, and granulocyte–macrophage progenitors (GMPs) in DON‐treated mice (Figure 1G,H). Collectively, these results demonstrate that DON profoundly disrupts the hierarchical organization and homeostasis of the hematopoietic system.

### 2.2 DON Exposure Impairs BM Erythroid Commitment and Differentiation

**Figure 2:**
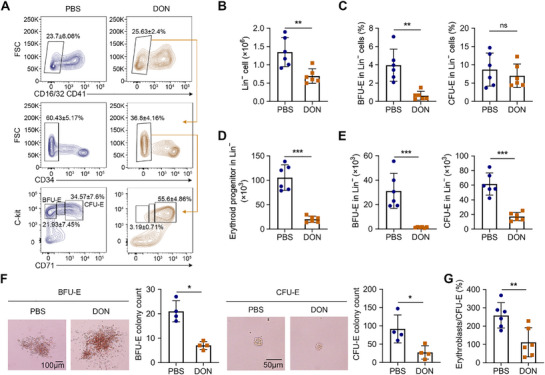
DON inhibits the differentiation of erythroid progenitors. A). Representative plots of flow cytometry analyses of BFU‐E and CFU‐E cells in mice BM. B). Number of Lin^−^ cells in the mice BM on day 1 following treatment with PBS or DON; *n* = 6 mice/group. C). Percentage of BFU‐E and CFU‐E cells in Lin^−^ cells; *n* = 6 mice/group. D). Number of erythroid progenitor cells; *n* = 6 mice/group. E). BFU‐E and CFU‐E cells in the mice BM on day 1 following treatment with PBS or DON; *n* = 6 mice/group. F). Left: Representative images of mice BFU‐E (Scale bar = 100 µm) and CFU‐E (Scale bar = 50 µm). Right: BFU‐E and CFU‐E colonies formed by plated BM cells of DON‐treated and control mice; *n* = 4 mice/group. G). The transformation ratio of CFU‐E to erythroblasts; *n* = 6 mice/group. Data are presented as mean ± SD. Comparisons between two groups were performing using unpaired two‐tailed Student's *t*‐test or Mann‐Whitney test. Significance was set at ^*^p < 0.05, ^**^p < 0.01, and ^***^p < 0.001.

Erythroid progenitors are pivotal for mass RBC production because of their high proliferation and effective differentiation abilities. Thus, we characterized the effects of DON on erythroid commitment using flow cytometry analyses of defined erythroid progenitor populations isolated from the BM (**Figure** **2**A). DON treatment drastically reduced the total Lin^−^ cell number in the BM (Figure 2B). Although the population of CD71^−/low^ BFU‐E cells within Lin^−^ cells decreased significantly, the percentage of CD71^high^ CFU‐E cells remained comparable to that of the controls (Figure 2C). However, absolute quantification demonstrated a severe reduction in c‐kit^+^ erythroid progenitor cells containing both BFU‐E and CFU‐E populations owing to the overall reduction in BM Lin^−^ cells (Figure 2D,E). Strikingly, cell cycle analysis demonstrated profound G1 phase arrest in erythroid progenitor cells (Figure S2, Supporting Information). Functional assessment using colony‐forming assays in semi‐solid MethoCult medium revealed markedly impaired erythroid colony forming capacity in DON‐treated BM, with significant reductions in both BFU‐E‐ and CFU‐E colonies (Figure 2F). This differentiation blockade was further evidenced by decreased erythroblasts versus CFU‐E ratios calculated from the flow cytometry data (Figure 2G). These findings demonstrate that DON exposure compromises erythroid lineage commitment and the colony‐forming abilities of erythroid progenitor cells, indicating a cellular mechanism for the observed anemia.

### 2.3 DON Impairs Terminal Erythropoiesis Through Cell Cycle Arrest in Mice

**Figure 3:**
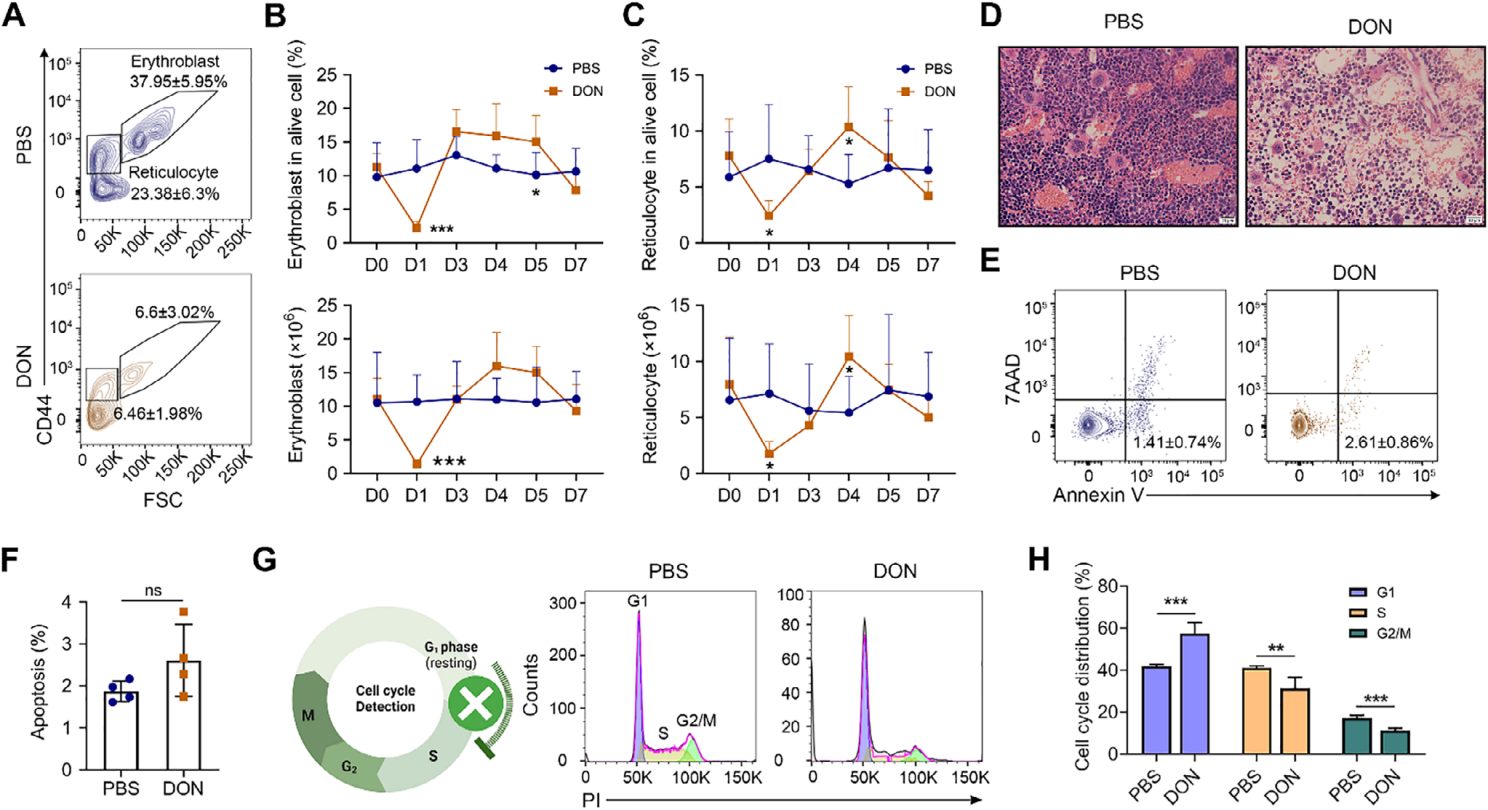
DON impairs terminal erythropoiesis in mice. A). Representative plots of mice BM erythropoiesis on day 1 following treatment with PBS and DON. B). Trends in erythroblast percentage and counts in PBS and DON‐treated mice; *n* = 4–12 mice/group. C). Trends in reticulocyte percentage and counts in PBS and DON‐treated mice; *n* = 4–12 mice/group. D). Representative images of hematoxylin and eosin staining of BM sections obtained from PBS and DON‐treated mice. Scale bar = 20 µm. E). Representative flow cytometric gating strategy of apoptotic cells in the mice BM erythroid cells through the expressions of 7AAD and Annexin V. F). Quantification analysis of apoptotic cells in the mice BM Ter119^+^ cells; *n* = 4 mice/group. G). Left: Schematic of cell cycle arrest. Right: Representative plots of the cell cycle distribution of Ter119⁺ BM cells from mice treated with PBS and DON. H). Quantification analysis of cell cycle distribution of the mice BM Ter119^+^ cells; *n* = 6 mice/group. Data are presented as mean ± SD. Comparisons between two groups were performed using the unpaired two‐tailed Student's *t*‐test or Mann‐Whitney test. Significance was set at ^*^p < 0.05, ^**^p < 0.01, and ^***^p  0.001.

To specifically determine whether DON‐induced anemia results from defective erythroid development, we analyzed erythroblasts and reticulocytes using the surface markers Ter119 and CD44 (**Figure** **3**A). Both the percentage and absolute number of erythroblasts and reticulocytes declined significantly on day 1 following DON treatment, with parallel recovery trends (Figure 3B,C), indicating impaired BM terminal erythropoiesis. Hematoxylin and eosin (H&E) staining of BM sections further confirmed the reduction in hematogenic activity (Figure 3D). The impaired BM erythropoietic activity is compensated by the spleen as demonstrated by mild splenomegaly and increased erythropoiesis in the spleen (Figure S3A–D, Supporting Information).

In addition to erythroid suppression, we observed a decrease in the number of peripheral white blood cells in the DON‐treated group compared with that in the controls (Figure S4A, Supporting Information). Flow cytometry analyses revealed that the number of granulocytes, T cells, B cells, and macrophages in the BM of DON‐treated mice decreased (Figure S4B–D, Supporting Information). Interestingly, while DON exposure induced apoptosis in non‐erythroid lineages (Figure S4E, Supporting Information)—consistent with previous studies^[^ ^10^, ^11^, ^27^ ^]^—erythroid precursors showed no significant increase in apoptosis (Figure 3E,F). Consistent with the cell cycle analysis of erythroid progenitor cells, erythroid cells exhibited profound G1 phase arrest (Figure 3G,H, Figure S5A,B, Supporting Information), indicating that DON impairs erythropoiesis primarily through cell cycle blockade rather than through apoptosis.

### 2.4 DON Compromises Human Erythropoiesis with Stage‐Specific Effects

**Figure 4:**
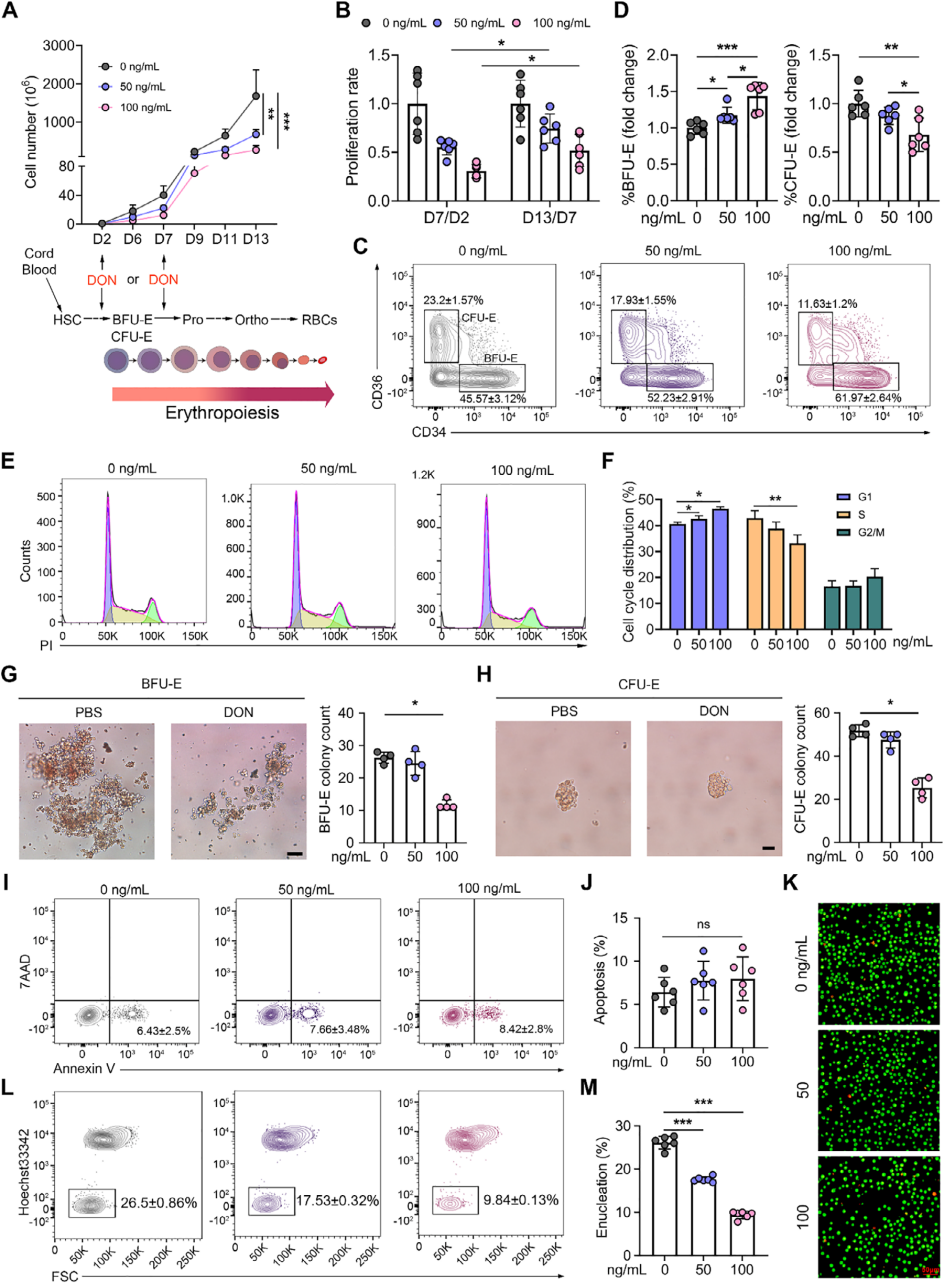
DON delays human erythroid progenitor differentiation. A). Growth curves of erythroid cells treated with DON (0, 50, and 100 ng mL^−1^) from day 2; *n* = 6. B). The ratio of proliferation changes between D7 and D2 and between D13 and D7. *n* = 6. C). Representative flow cytometry analyses plots of the human erythroid progenitor treated with PBS and different concentrations of DON on day 5. D). Rate of BFU‐E and CFU‐E cells on day 5 in the CD34^+^ cell culture; *n* = 6. E). Representative plots of the cell cycle distribution of human erythroid progenitor cells treated with PBS and DON (0, 50, and 100 ng mL^−1^). F). Quantification analysis of cell cycle distribution; *n* = 4. G). Left: Representative images of human BFU‐E. Right: Number of BFU‐E colonies formed by 200 plated cells cultured on day 14; *n* = 4. Scale bar = 50 µm. H. Left: Representative images of human CFU‐E. Right: Number of CFU‐E colonies formed by 200 plated cells cultured on day 7; *n* = 4. Scale bar = 20 µm. I). Representative plots of human apoptotic erythroid cells. J). Quantification analysis of human apoptotic erythroid cells on day 13 in the presence of PBS and DON (0, 50, and 100 ng mL^−1^); n = 6. K). Representative immunofluorescence images of erythroid progenitor cells using acridine orange/ethidium bromide staining. Scale bar = 50 µm. L). Representative plots of human enucleated erythroid cells. M). Quantification analysis of enucleated cells in human erythroid cells on day 15 in the presence of PBS and DON (0 and 100 ng mL^−1^); *n* = 6. Data are presented as mean ± SD. ANOVA with Tukey's *post hoc* test was used to calculate statistical significance among multiple groups. Significance was set at ^*^p < 0.05, ^**^p < 0.01, and ^***^p < 0.001.

To evaluate the conserved effects of DON on mammalian erythropoiesis, we differentiated human cord blood (CB) CD34^+^ cells into erythroid cells using a three‐phase erythroid culture system.^[^ ^22^, ^26^ ^]^ Growth curves showed that cell growth was significantly inhibited by DON treatment in a dose‐dependent manner (**Figure** **4**A). Furthermore, a stage‐specific analysis of the proliferation revealed greater DON sensitivity during early (D2–D7) versus terminal (D7–D13) erythropoiesis (Figure 4B). First, we examined the differentiation of CMPs, MEPs, and GMPs on day 4, and we observed that these lineages were all affected by DON treatment (Figure S6A,B, Supporting Information) with significant apoptosis (Figure S6C, Supporting Information). Next, we explored the effects of DON on early‐stage human erythropoiesis. Compared with the control group, the percentage of BFU‐E (IL3R^−^GPA^−^CD34^+^CD36^−^ population) significantly increased, whereas the proportion of CFU‐E (IL3R^−^GPA^−^CD34^−^CD36^+^ population) markedly decreased on day 5 (Figure 4C,D), indicating blocked differentiation of early‐stage erythropoiesis. The counts of both human BFU‐E and CFU‐E cultured in vitro were significantly reduced compared with controls (Figure S7A, Supporting Information), consistent with our in vivo findings in mice described above. Cell cycle analysis confirmed G1 phase arrest in human erythroid progenitors following DON exposure (Figure 4E,F). Colony‐forming assays revealed a significant reduction in the number of colonies formed by BFU‐E and CFU‐E cells in the DON‐treated groups (Figure 4G,H), indicating the impaired colony‐forming capacity of erythroid progenitors. Further, the effects of DON on terminal erythroid differentiation were investigated. Monitoring of glycophorin A (GPA) expression, which indicates the transition from CFU‐E cells to proerythroblasts,^[^ ^26^, ^28^ ^]^ showed no difference in the percentage of GPA^+^ cells on days 9 and 15, with or without DON treatment (Figure S7B,C, Supporting Information). We further conducted a detailed evaluation of terminal erythroid differentiation utilizing Band3 and α4 integrin as surface markers.^[^ ^26^ ^]^ The results showed no discernible differences between the DON‐treated and control groups (Figure S7B, Supporting Information). In addition, DON did not induce apoptosis in human erythroid cells (Figure 4I–K). Erythroblast enucleation is an essential step in erythropoiesis that creates extra space for cells to accommodate HGB for oxygen transport and provides cells with better deformability to pass through capillaries.^[^ ^29^ ^]^ We assessed erythroblast enucleation on day 15 using Hoechst33342 staining and found that DON had a significant inhibitory effect on this process (Figure 4L,M). To confirm the stage‐specific effects of DON on erythroid differentiation, we treated cultured erythroid cells with DON on day 7, when most cells were in the terminal erythropoietic stage. Late‐stage exposure recapitulated proliferative suppression (Figure S7D, Supporting Information). Notably, DON did not affect the proportion of GPA^+^ cells or induce apoptosis in human terminal erythroblasts (Figure S7E,F, Supporting Information); however, DON consistently impaired erythroblast enucleation (Figure S7G, Supporting Information). Collectively, these findings demonstrate that DON severely compromises human erythropoiesis, particularly during early differentiation and enucleation.

### 2.5 DON Perturbs the Proteome Profile without Corresponding Transcriptional Alterations

To elucidate the mechanisms underlying DON‐induced erythropoietic defects, we performed RNA sequencing (RNA‐seq) on day 6 human erythroid progenitor cells. Principal component analysis (PCA) validated the reliability of the sampling and sequencing methods (Figure S8A, Supporting Information). Based on pairwise comparisons, 135 differentially expressed genes (36 upregulated and 99 downregulated) were identified between the two samples (Figure S8B–D, Supporting Information). Gene set enrichment analysis (GSEA) revealed significant enrichment of the “ribosome biogenesis” pathway in DON‐treated cells (Figure S8E, Supporting Information), aligning with prior reports of DON‐induced ribosomal damage.^[^ ^30^, ^31^, ^32^, ^33^, ^34^ ^]^

**Figure 5:**
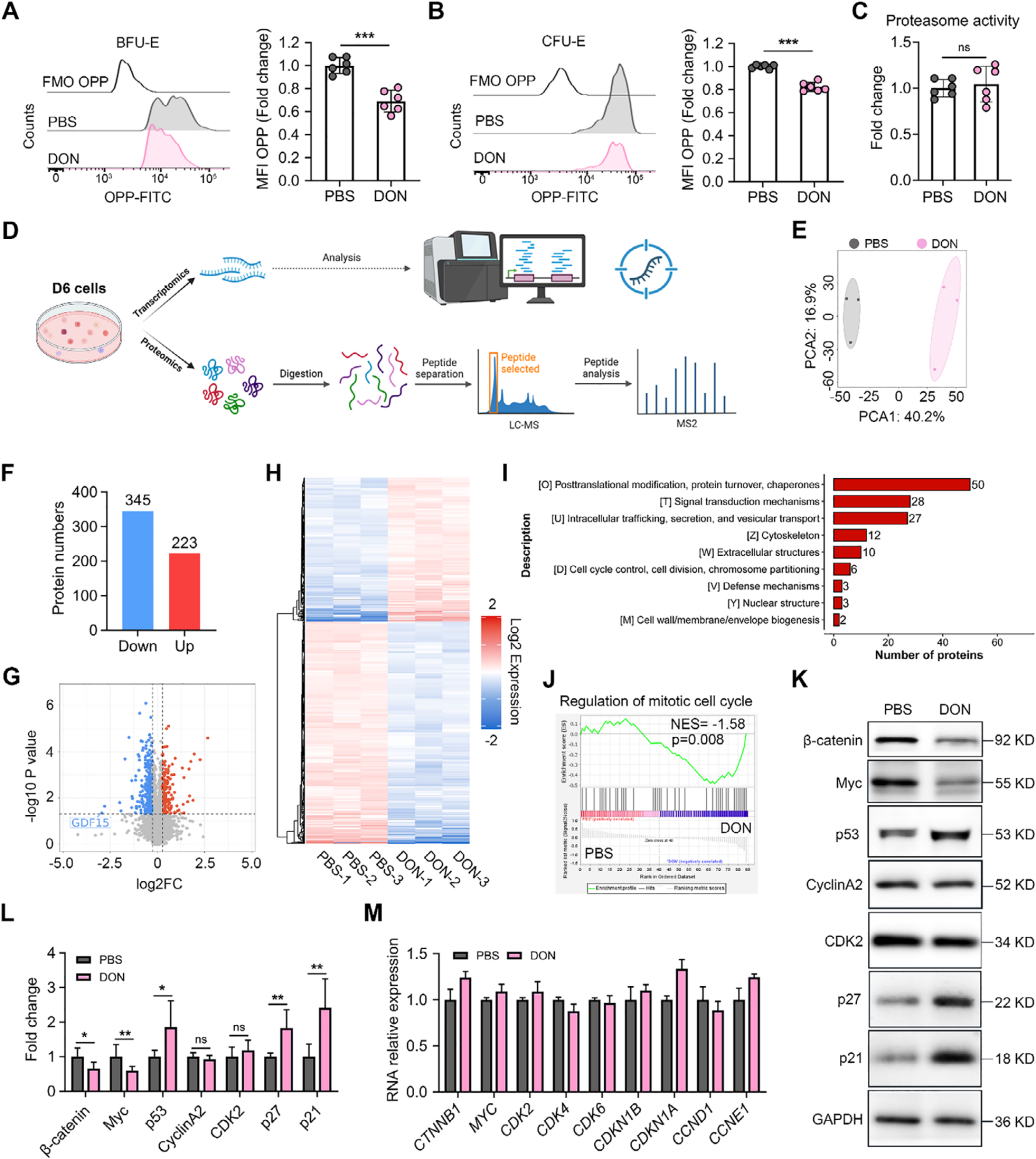
DON inhibits the cell cycle along with the post‐transcriptional translation. Representative flow cytometry profiles and quantitative analyses of protein synthesis in A). BFU‐E and B). CFU‐E; *n* = 6. C). Quantitative analysis of intracellular proteasome activity level on day 6; *n* = 6. D). Flowchart depicting cell collection, followed by RNA and protein sequencing. E). Principal component analysis of all proteomic samples, with the first and second components being shown. F). Number of differentially expressed proteins in PBS‐ and DON‐treated erythroid progenitor cells. G). Volcano plots showing proteomic changes in PBS‐ and DON‐treated erythroid progenitor cells. H). Heatmap of the differentially expressed proteins. I). Clusters of orthologous groups analysis of the differentially expressed proteins showing the ranks of downregulated categories associated with cellular processes and signaling. J). Gene set enrichment analysis performed on proteomics data revealed the regulation of mitotic cell cycles. K). Representative western blotting images and L). quantification of β‐catenin, Myc, p53, CyclinA2, CDK2, p27, and p21 levels in human erythroid progenitor cells cultured on day 6 with PBS or DON treatment, with GAPDH used as a loading control; *n* = 3–7. M). The qRT‐PCR analysis and quantification of the mRNA expression levels of *CTNNB1, MYC, CDK2, CDK4, CDK6, CDKN1B, CDKN1A, CCND1*, and *CCNE1* in human erythroid progenitor cells cultured on day 6 with PBS or DON treatment; *n* = 3. Data are presented as mean ± SD. Comparisons between two groups were performed using the unpaired two‐tailed Student's *t*‐test or Mann‐Whitney test. Significance was set at ^*^p < 0.05, ^**^p < 0.01, and ^***^p < 0.001.

Given the minimal transcriptional changes and ribosomal targeting, we hypothesized that DON primarily disrupts protein synthesis beyond transcriptional regulation. We observed significant reductions in protein synthesis rates in BFU‐E and CFU‐E cells following DON exposure using an O‐propargyl‐puromycin incorporation assay (**Figure** **5**A,B), during which no significant change in proteasome activity was detected (Figure 5C). These findings suggest that DON may impair erythropoiesis by dysregulating the synthesis of certain crucial proteins rather than through transcriptional control or protein degradation. We further performed a proteomic analysis of day 6 human erythroid progenitor cells (Figure 5D). PCA revealed a clear separation between the DON‐treated and control groups, demonstrating a unique expression signature of DON‐treated erythroid progenitors (Figure 5E). In contrast to transcriptional stability, there were dramatic proteomic alterations, with 568 differentially expressed proteins (223 upregulated and 345 downregulated) meeting significance thresholds (fold change > 1.2, *p* < 0.05) (Figure 5F,G). A hierarchical clustering heatmap of the differentially expressed proteins is shown in Figure 5H. Clusters of orthologous groups and GSEA analyses demonstrated a particular enrichment of cell cycle regulatory proteins among these changes (Figure 5I,J). Then we examined the expression of a panel of proliferation and cell cycle related genes. For this, we focused on β‐catenin, Myc and p21 because the role of the β‐catenin–Myc–p21 axis in cell‐cycle control has been established by previous studies. Specifically, it has been reported that β‐catenin promotes the transition of the cell cycle from the G0/G1 phase to the S phase by activating the transcription of cell‐cycle genes including *Myc*.^[^ ^35^, ^36^ ^]^ The role of the Myc–p21 axis has been demonstrated: Myc normally inhibits p21 expression, and the absence of Myc relieves its inhibition effect, resulting in p21 upregulation. This, in turn, causes cell cycle arrest at the G0/G1 phase and suppresses proliferation.^[^ ^37^, ^38^ ^]^ Western blotting confirmed a 50% reduction in β‐catenin and Myc, accompanied by increased p21 and p27 levels (Figure 5K,L), corroborating the G1‐phase cell cycle arrest observed in DON‐treated erythroid cells. Intriguingly, there were no significant alterations in mRNA levels of these genes (Figure 5M). These findings establish that DON primarily disrupts erythropoiesis through post‐transcriptional mechanisms, impairing translation of critical cell cycle regulators while maintaining transcriptional homeostasis.

### 2.6 GDF15 Reverses DON‐Induced Erythroid Dysfunction

**Figure 6:**
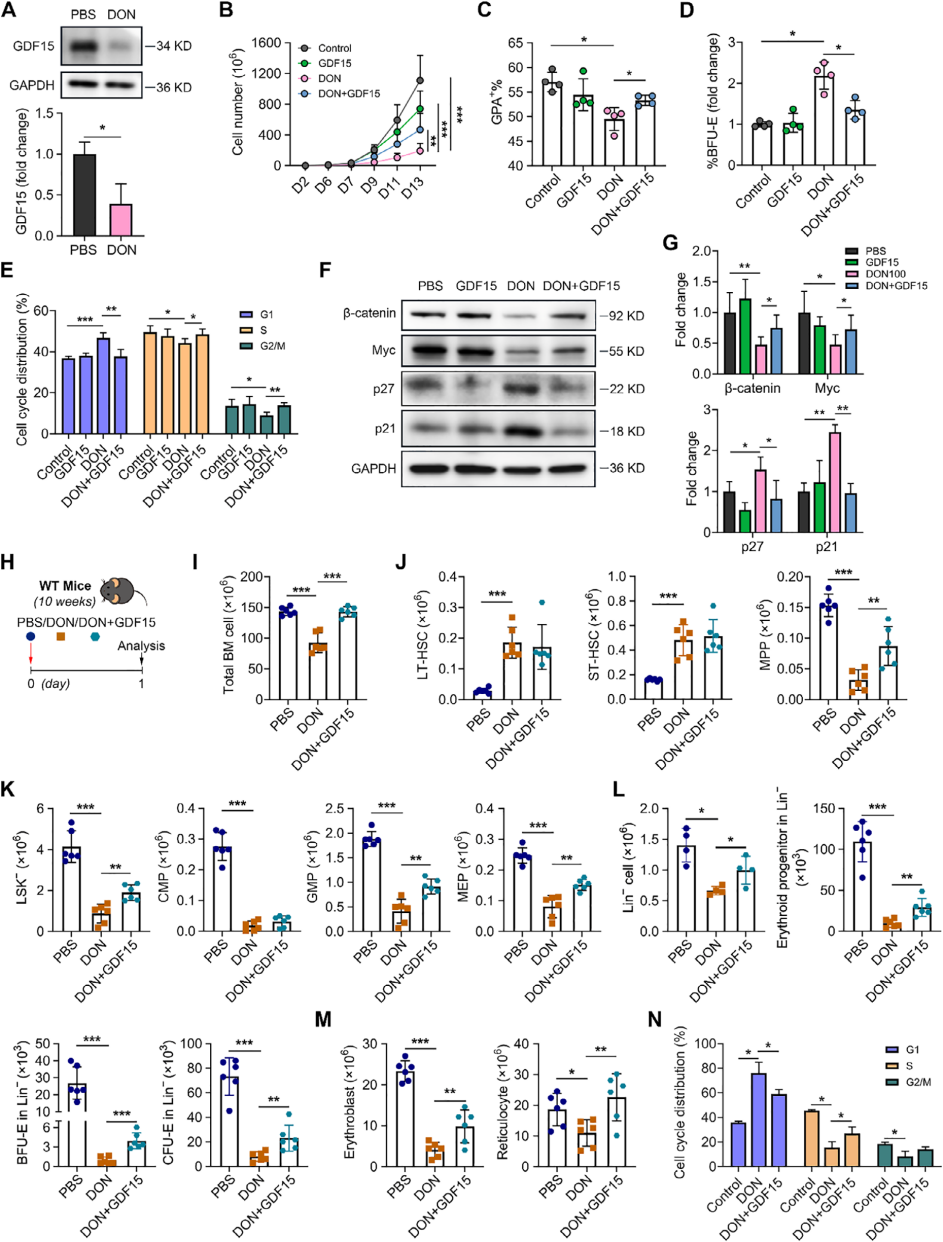
GDF15 restores the adverse in vitro and in vivo effects of DON poisoning. A). Representative western blotting images and quantification of GDF15 levels in human erythroid progenitor cells cultured on day 6 with PBS or DON treatment, with GAPDH used as a loading control; *n* = 4. B). Growth curves of erythroid cells treated with GDF15 (50 ng mL^−1^), DON (100 ng mL^−1^), and GDF15 combined with DON from day 2; *n* = 7. C). The percentage of GPA^+^ cells on day 6; *n* = 4. D). Rate of BFU‐E cells on day 6 in the CD34^+^ cell culture; *n* = 4. E). Quantification analysis of cell cycle distribution; n = 4. F). Representative western blotting images and G). quantitation of β‐catenin, Myc, p27, and p21 in human erythroid progenitor cells cultured on day 6, with GAPDH used as a loading control; *n* = 4–6. H). Schematic model of GDF15‐treated mice. I). Quantification of total BM cells; *n* = 6 mice/group. The number of J). LT‐HSC, ST‐HSC, and MPP; K). LSK^−^, CMP, GMP, and MEP; L). Lin^−^ cells, erythroid progenitor cells, BFU‐E, and CFU‐E; and M). erythroblasts and reticulocytes in the BM on day 1 following treatment with PBS, or DON, or DON combined with GDF15 in mice; *n* = 4–6 mice/group. N). Quantification analysis of the cell cycle distribution of murine BM erythrocytes; *n* = 3–6. Data are presented as mean ± SD. ANOVA with Tukey's *post hoc* test was used to calculate statistical significance among multiple groups. Significance was set at ^*^p < 0.05, ^**^p < 0.01, and ^***^p  0.001.

Finally, we attempted to establish a strategy for alleviating DON‐induced hematopoietic disorders. Based on bioinformatics analysis, we focused on GDF15 as it was identified to be the most significantly downregulated protein (Figure 5G) despite stable mRNA expression (Figure S8F, Supporting Information). Additionally, GDF15 has been shown to play an important role in the regulation of erythroid progenitor differentiation.^[^ ^39^, ^40^ ^]^ Western blotting validated decreased levels of GDF15 (**Figure** **6**A). We also performed shRNA‐mediated knockdown of GDF15 in erythroid cells. This led to reduced proliferation, and G0/G1 phase cell‐cycle arrest, recapitulating the phenotype observed in DON‐treated cells (Figure S9A–C, Supporting Information).To test the therapeutic potential of GDF15, we initially supplemented DON‐treated human erythroid cultures with 50 ng mL^−1^ GDF15 (from day 2) and observed that GDF15 significantly increased erythroid cell numbers, restored BFU‐E levels to normal, and increased GPA^+^ cell percentages (Figure 6B–D), resolving the blockade of early‐stage erythropoiesis caused by DON. Notably, GDF15 alleviated G1‐phase arrest (Figure 6E) and restored the expressions of β‐catenin, Myc, p27, and p21, to control levels (Figure 6F,G). To examine the effects of GDF15 supplementation in vivo, we treated the mice with 150 µg kg^−1^ GDF15 following exposure to DON (Figure 6H) and found that GDF15 effectively restored BM cellularity (Figure 6I) and hematopoietic hierarchy, particularly rescuing MPP, GMP, and MEP populations (Figure 6J,K). Moreover, BM erythroid cells, including progenitors, erythroblasts, and reticulocytes, showed significant recovery (Figure 6L,M), accompanied by resolution of the G1 phase blockade (Figure 6N). These results established GDF15 as a potent therapeutic agent capable of reversing DON‐induced erythropoietic suppression at both the cellular and molecular levels.

## 3 Discussion

Contamination of food supplies by DON poses a persistent threat to global health, with hematotoxicity as a clinically significant consequence.^[^ ^15^, ^41^, ^42^ ^]^ Our study systematically delineates the mechanisms underlying DON‐induced anemia, which includes disrupted erythroid commitment and differentiation through ribosomal stress–mediated G1 cell cycle arrest. By integrating murine models, human primary cell cultures, and multi‐omics analyses, we demonstrated that it exerted erythropoietic toxicity in an apoptosis‐independent manner, and this primarily stems from the translational suppression of GDF15 and subsequent dysregulation of the β‐catenin–Myc–p21 axis. Furthermore, we identified GDF15 as a novel therapeutic target for reversing DON‐induced erythropoietic failure.

Consistent with prior reports in chickens, rats, and piglets,^[^ ^42^, ^43^, ^44^ ^]^ DON exposure in mice caused a rapid decline in peripheral RBC counts, HGB levels, HCT levels, and reticulocytes. While previous in vivo studies attributed these effects to BM suppression,^[^ ^15^ ^]^ our data revealed a considerably nuanced mechanism: DON triggers the compensatory expansion of HSCs but severely depletes MPPs and downstream lineages (CMPs, GMPs, and MEPs). This disruption extends to the erythroid progenitors and erythroblasts, culminating in anemia. Previous in vitro studies have mainly been conducted using immortalized cell lines,^[^ ^10^, ^12^, ^31^, ^45^ ^]^ which have certain limitations, such as an inability to fully recapitulate the behavior of normal cells and a tendency to yield relatively fragmented results limited to specific time points. In this study, we employed an in vitro primary human erythroid differentiation system^[^ ^22^, ^26^ ^]^ that demonstrated conserved erythropoietic defects, with DON preferentially blocking early differentiation evidenced by BFU‐E accumulation alongside CFU‐E depletion. The increased sensitivity of early erythropoiesis suggests that rapidly dividing progenitors are more vulnerable to DON‐induced translational arrest. Interestingly, we found that DON disrupts the differentiation of hematopoietic progenitor cells and erythroid cells through different pathways. Therefore, we will continue to investigate the molecular mechanisms responsible for the reduction of hematopoietic progenitor cells to identify additional targets for addressing the challenges in treating DON poisoning.

The discovery that DON preferentially targets protein synthesis over transcription in erythroid progenitors explains its potent disruption of erythropoiesis despite minimal changes in mRNA abundance. Our findings are consistent with prior reports on DON ribosomal targeting.^[^ ^13^, ^30^ ^]^ Additionally, our study extends this paradigm by linking ribosomal stress to specific erythropoietic defects. The near absence of transcriptional changes is in sharp contrast to the profound proteomic alterations, particularly in cell cycle pathways. This discordance suggests that the DON‐induced inhibition of peptidyl transferase activity ^[^ ^33^ ^]^ selectively impairs the translation of short‐lived proteins, such as Myc and β‐catenin,^[^ ^45^ ^]^ which are essential for progenitor proliferation. Meanwhile, when ribosome biogenesis is disrupted, it prevents p53 from being degraded, thereby stabilizing and accumulating p53. Activated p53 transcriptionally activates downstream target genes, among which p21, as a direct transcriptional target of p53, induces cell cycle arrest at the G1 phase, making cells unable to enter the S phase.^[^ ^46^, ^47^ ^]^ The specificity of this mechanism explains the lineage‐restricted cytotoxicity observed. Ribosome profiling combined with qRT‐PCR or RNA‐seq may further elucidate the translational defects observed in DON‐treated erythroid cells and represents a promising direction for future studies. This particularity reminds us of Diamond‐Blackfan anemia (DBA), a genetic blood disorder in which the majority of mutations affect ribosomal proteins and the erythroid lineage is selectively perturbed.^[^ ^48^ ^]^ The reduction in ribosome levels may profoundly alter the translation of a select subset of transcripts that are normally highly translated and have short/unstructured 5′ untranslated regions as compared with other transcripts.^[^ ^49^ ^]^ Findings regarding DON‐induced ribosomal stress–mediated cell cycle arrest, responsible for erythroid progenitor cell disorders, may contribute to understanding of the underlying mechanisms of ribosomal functional defects that selectively impact erythroid development in DBA.

Stable translational regulation ensures the normal differentiation of erythroid progenitor cells through the mTORC1‐TOP‐like motif‐mitochondrial protein synthesis axis and serves as a key regulatory node in erythroid maturation. ^[^ ^50^ ^]^ In addition to perturbations in cell cycle–related proteins, our proteomic data revealed DON‐associated alterations across other functional categories, including several metabolic pathways and various proteins related to the structure and function of mitochondrial matrix. These findings suggest that DON‐induced ribosomal targeting may broadly affect erythroid proteome remodeling beyond cell cycle control. We will further explore in future research whether these functional changes are also important factors in DON targeting ribosomes to disrupt erythropoiesis.

Central to this study was the discovery of GDF15 as a mechanistic node and therapeutic target. GDF15, a cytokine within the transforming growth factor‐β family, plays crucial roles in metabolism, angiogenesis, and regeneration.^[^ ^51^, ^52^, ^53^, ^54^ ^]^ Emerging research has identified its important role in normal and stress erythropoiesis.^[^ ^39^, ^40^ ^]^ Our discovery that DON depletes GDF15 protein without altering its transcription unveils a pivotal node in DON toxicity. GDF15 supplementation reversed DON‐induced defects both in vitro and in vivo. Mechanistically, GDF15 normalized the dysregulation in β‐catenin–Myc–p21 axis, suggesting that it either bypasses ribosomal blockade or stabilizes the translation of short‐lived cell cycle proteins. Additionally, given the involvement of GDF15 in tissue repair and inflammation,^[^ ^54^, ^55^ ^]^ its rapid decline following DON exposure may exacerbate erythropoietic failure by impairing progenitor resilience. Moreover, recent studies have demonstrated that GDF15 plays a crucial role in the proliferation and metabolic reprogramming of stress erythroid progenitors, in part through the transcriptional regulation of key erythroid transcription factors such as GATA‐1 and KLF‐1.^[^ ^56^ ^]^ In our study, we observed that GDF15 deficiency led to cell cycle arrest at multiple stages of erythroid differentiation. Mechanistically, we found that this arrest is associated with downregulation of the β‐catenin–Myc–p21 signaling axis, which is known to regulate cell cycle progression. Previous studies have shown that GDF15 modulates cell cycle and proliferation by altering p21 expression through the PI3K/AKT, MAPK/ERK, and STAT3 pathways.^[^ ^57^, ^58^ ^]^ Given that GATA‐1 and KLF‐1 are also involved in cell cycle control during erythropoiesis, it is possible that GDF15 modulates the erythroid cell cycle both directly through the β‐catenin pathway and indirectly via transcriptional control of these lineage‐specific factors. Further investigation into the interaction between these pathways may provide deeper insights into how GDF15 orchestrates erythroid homeostasis under stress conditions. Furthermore, GDF15 supplementation could mitigates apoptosis on non‑erythroid cells, indicating that GDF15 may exert lineage‑specific protective effects through distinct mechanisms, highlighting its therapeutic potential against the diverse hematopoietic defects caused by DON. The efficacy of GDF15 in restoring BM progenitors and erythroid output, coupled with the recovery of cell cycle progression, suggests that GDF15 could serve as a biomarker of DON exposure severity as well as a therapeutic agent, particularly in acute poisoning scenarios. Given the frequent occurrence of food poisoning cases involving DON, further clinical studies are warranted to explore the role of GDF15 in counteracting DON poisoning and to assess the feasibility of rapid therapeutic interventions.

In conclusion, our study revealed, for the first time, the mechanism of hematopoietic damage caused by DON poisoning and identified GDF15 as a key mediator and therapeutic target. These findings advance the mechanistic understanding of mycotoxin toxicity and bridge fundamental ribosome biology with practical interventions for food safety, offering new strategies to mitigate the global health burden of DON contamination.

## 4 Experimental Section

### Reagents

DON standard (CAS No. 51481‐10‐8) with purity ≥98% was purchased from Absin Bioscience Inc.; it was dissolved in phosphate buffer saline (PBS) and stored at −20 ℃. The final concentrations of the solvents used in the cell culture were 50 and 100 ng mL^−1^, whereas in the mice experiment, it was 10 mg kg^−1^.^[^ ^59^, ^60^, ^61^, ^62^ ^]^ Human GDF15/MIC‐1 recombinant protein (120‐28C), with a purity of ≥95%, was purchased from Thermo Fisher Scientific; it was dissolved in ddH_2_O and stored at −20 ℃. The final concentration of solvent used in the cell culture was 50 ng mL^−1^, whereas in the mice experiment, it was 150 µg kg^−1^.

### Animal Experiments

Wild‐type C57BL/6 male mice (8 weeks) were purchased from SPF (Beijing) Biotechnology Co., Ltd. Animals were housed in polypropylene cages within a separate ventilated room that replicated the environmental conditions at SPF: temperature, 21 ± 1 ℃; relative humidity, 40%–70%; and regular light/dark cycle, 12:12 with ad libitum access to water and food. The diet complied with the national standards GB13078 and GB14924.2. Animal experiments were performed in accordance with the Animal Ethics Committee of Zhengzhou University (ZZUIRB2024‐59), the U.K. Animals (Scientific Procedures) Act (1986), and the associated guidelines of the EU Directive 2010/63/EU for animal experimentation. Mice were randomly divided into the control and DON treatment groups. The DON treatment group was intraperitoneally injected with a dose of 10 mg/kg DON on day 0 and euthanized on days 1, 3, 4, 5, and 7 separately for further analysis. Control group mice received only the vehicle.

### Cell Culture

Human CB samples were collected from the People's Hospital of Zhengzhou (approval number: ZZUIRB2023‐242); informed consent was obtained from all individuals. CD34^+^ cells were isolated from human CB through positive selection using a magnetic activated cell sorting system, according to the manufacturer's instructions. The purity of isolated CD34^+^ cells ranged from 95% to 98%. Cells were cultured in Iscove's Modified Dulbecco's medium containing 8% heat‐inactivated fetal bovine serum (FBS), 2% human peripheral blood plasma, 10 µg mL^−1^ insulin, 3 IU mL^−1^ heparin, 10 µg mL^−1^ stem cell factor, 1 µg mL^−1^ interleukin‐3, 3 IU mL^−1^ erythropoietin, 200 µg mL^−1^ transferrin, and 1% penicillin‐streptomycin in a humidified incubator (37 ℃; 5% CO_2_). The cell culture procedure comprised three phases. In the first phase (D0–D7), CD34^+^ cells were cultured in the medium at a concentration of 10^5^/mL as mentioned above. In the second phase (D7–D11), IL‐3 was omitted from the culture medium. In the third phase (D11–D15), SCF and IL‐3 were excluded from the culture medium.^[^ ^26^ ^]^

### Examination of Blood Parameters

Mice blood samples were collected from the suborbital vein and diluted with normal saline at a ratio of 1:6, followed by examination using the ADVIA2120i hematology system (Siemens).

### Histology and Immunohistochemistry

Mice bone sections were cut from paraffin‐embedded tissue. Following decalcification, morphological changes in the BM were detected using the H&E staining kit (Servicebio). Images were captured using a light microscope (Olympus).

### Flow Cytometry Analyses

For the mouse BM, cells were flushed with Buffer I (PBS + 2% FBS + 2 mm ethylenediaminetetraacetic acid) and smashed through a cell strainer into a centrifuge tube, as previously described.^[^ ^25^ ^]^ Depletion of the mouse BM lineage^+^ cells and flow cytometry analyses of hematopoietic stem and progenitor cell (HSPC) development, erythropoiesis, granulocytes, macrophages, T and B cells were performed in Supplementary Materials and Methods. For human erythropoiesis, cultured erythroid cells were analyzed through surface expressions of GPA, IL3R, CD34, CD36, Band3, and a4‐integrin. The antibodies were performed in Supplementary Materials and Methods. Hoechst33342 was used to detect the rate of erythroblast enucleation.^[^ ^23^ ^]^ Annexin V kit (eBioscience #88‐8103‐74) was used for apoptosis detection.^[^ ^22^ ^]^ Propidium iodide (Solarbio #P8080) was used for cell cycle detection according to the manufacturer's protocol.

### Colony Formation Assay

Mice BM cells were plated at a density of 50000 cells per 1 mL of MethoCult M3434 (Stemcell #0 3434) medium for the BFU‐E colony formation assay, and in MethoCult M3334 (Stemcell #0 3334) medium for the CFU‐E colony formation assay. They were incubated at 37 ℃ in a humidified atmosphere incubator with 5% CO_2_. After 3 days, mouse CFU‐E completed differentiation (into terminal erythroid cells) in M3334 medium, and after 7 days, mouse BFU‐E completed differentiation in M3434 medium.^[^ ^63^ ^]^ For the human samples, day 6 in vitro cultured erythroid cells were plated at a density of 200 cells per 1 mL of MethoCult H4434 (Stemcell #0 4434) medium for the BFU‐E colony formation assay or MethoCult H4330 (Stemcell #0 4330) medium for the CFU‐E colony formation assay. CFU‐E and BFU‐E colonies were counted after 7 and 14 days, separately.^[^ ^64^, ^65^ ^]^

### Acridine orange/ethidium bromide (AO/EB) Staining

CB‐derived erythroblasts were stained with an AO/EB kit (Bestbio #BB20071) according to the manufacturer's protocol, and fluorescence signals were imaged using a laser scanning confocal microscope (ZEISS).

### Western Blot Analysis

The total protein from cultured human erythroblasts was extracted using radioimmunoprecipitation assay solution. Protein samples were separated using 10% SDS‐PAGE, followed by incubation with specific antibodies including anti‐β‐catenin (66379‐1‐Ig), anti‐CDK2 (PTM‐5121), anti‐p21 (PTM‐6510), anti‐p27 (PTM‐6471), anti‐Myc (5605S), anti‐Cyclin A2 (66391‐1‐lg), anti‐p53 (10442‐1‐AP) and anti‐GAPDH (GB15004), followed by incubation with HRP‐conjugated IgG antibodies. Immunoblotted proteins were visualized using an enhanced chemiluminescence system.^[^ ^66^ ^]^

### RNA‐seq and Bioinformatic Analysis

CB‐derived erythroblasts were treated with DON on day 2 and collected on day 6. Total RNA was extracted using the TRIzol reagent. Libraries were then constructed using the optimal dual‐mode mRNA library prep kit (BGI‐Shenzhen, China) according to the manufacturer's instructions. Transcriptome sequencing and analysis were performed by BGI Genomics Co. (Shenzhen, China).

### Proteomics Analysis

CB‐derived erythroblasts were treated with DON on day 2 and collected on day 6. Proteins were extracted using lysis buffer (1% sodium deoxycholate and 1% protease inhibitor), and the protein concentration was determined using a BCA kit. Proteomic sequencing and analysis were performed by PTM BioLab Co. (Hangzhou, China).

### Statistical Analysis

All the studies were randomized and blinded. Flow cytometry data were analyzed using FlowJo software. ImageJ software was used to analyze band signal intensities. Statistical analyses and removal of outliers were performed using GraphPad Prism 10.0. Data are presented as mean ± SD. Comparisons between two groups were performed using unpaired two‐tailed Student's *t*‐test (n ≥ 6) or Mann–Whitney test (n < 6). ANOVA with Tukey's *post hoc* test was used to calculate statistical significance among multiple groups. Significance was set at ^*^p < 0.05, ^**^p < 0.01, and ^***^p < 0.001. For normalized data, log10‐transformation was applied to reduce skewing; the back‐transformed means were reported for clarity.

### ARRIVE Statement

All animal experiments were carried out in accordance with the ARRIVE guidelines, the U.K. Animals (Scientific Procedures) Act (1986), and the associated guidelines of the EU Directive 2010/63/EU for animal experimentation.

## Conflict of Interest

The authors declare no conflict of interest.

## Author Contributions

Y.L. and P.J. contributed equally to this work. Y.L. and P.J. designed the experiments, performed the research, analyzed the data, and drafted the materials and methods. H.Z., B.L., J.C., Y.L., and L.Z. performed in vivo or in vitro experiments. H.Z. and Y.X. drafted the materials and methods. T.Z. and Y.Z. performed the bioinformatics analysis. J.Z., and X.A. analyzed the data and edited the manuscript. S.Z. conceived the subject, designed experiments, analyzed data and wrote the manuscript.

## Supporting information



Supporting Information

## Data Availability

The data that support the findings of this study are available from the corresponding author upon reasonable request.
