# Supplementary material for: GDF15 Ameliorates Deoxynivalenol‐Induced Anemia by Resolving Ribosomal Stress–Mediated Erythropoietic Arrest
Source: Adv Sci (Weinh). 2025 Sep 3;12(44):e09265. doi: 10.1002/advs.202509265 (PMC12667554; doi:10.1002/advs.202509265)
Supplement: Supplementary file 1 — Supporting Information [file ADVS-12-e09265-s001.docx]

**Supporting Information**

**GDF15 Ameliorates** **Deoxynivalenol-Induced Anemia by Resolving Ribosomal Stress-Mediated Erythropoietic Arrest**

Yan Li^a,#^, Peijun Jia^a,#^, jingxin Zhang^a^, Huan Zhang^a^, Bing Li^a^, Jiawei Chang^a^, Yating Li^a^, Longzhen Zhao^a^, Yazhe Zhen^a^, Tingting Zheng^a^, Yuanlin Xu^b^, Xiuli An^c,^, Shijie Zhang^a,*^

^a^ School of Life Sciences, Zhengzhou University, Zhengzhou, Henan 450000, China

^b^ Department of Internal Medicine, The Affiliated Cancer Hospital of Zhengzhou University & Henan Cancer Hospital, Zhengzhou, Henan 450008, China

^c^ Laboratory of Membrane Biology, New York Blood Center, New York, NY 10065, USA

*Corresponding author: [shijie-zhang@zzu.edu.cn](mailto:shijie-zhang@zzu.edu.cn) (Shijie Zhang)

**Supplementary** **Materials and Methods**

| REAGENT or RESOURCE | SOURCE | IDENTIFIER |
| --- | --- | --- |
| Antibodies | | |
| APC-Cy™7 Rat Anti-Mouse CD45 | BD | Cat# 557659 |
| APC-Cy™7 Rat Anti-CD11b | BD | Cat# 557657 |
| APC-Cy™7 Rat Anti-Mouse Ly-6G and Ly-6C | BD | Cat# 557661 |
| APC Rat Anti-Mouse CD44 | BD | Cat# 559250 |
| BV421 Anti-mouse TER-119/Erythroid Cells Antibody | Biolegend | Cat#116234 |
| CD11b Monoclonal Antibody (M1/70), Biotin | eBioscience | Cat#13-0112-85 |
| CD3e Monoclonal Antibody (145-2C11), Biotin | eBioscience | Cat#13-0031-85 |
| Biotin anti-mouse/human CD45R/B220 Antibody | Biolegend | Cat# 103204 |
| TER-119 Monoclonal Antibody (TER-119), Biotin | eBioscience | Cat#13-5921-85 |
| Anti-MoLy-6G/Ly6C | eBioscience | Cat#13-5931-85 |
| APC/Cyanine7 anti-mouse CD117 (c-kit) Antibody | Biolegend | Cat#105826 |
| APC anti-mouse CD34 Antibody | Biolegend | Cat#119310 |
| Brilliant Violet 421™ anti-mouse CD135 Antibody | Biolegend | Cat#135314 |
| Brilliant Violet 605™ anti-mouse Ly-6A/E (Sca-1) Antibody | Biolegend | Cat#108134 |
| Streptavidin | Biolegend | Cat# 405213 |
| Brilliant Violet 510™ anti-mouse CD16/32 Antibody | Biolegend | Cat#101333 |
| PerCP/Cyanine5.5 anti-mouse CD41 Antibody | Biolegend | Cat#133918 |
| BV421 Rat Anti-Mouse CD105 | BD | Cat#562760 |
| Brilliant Violet 421 anti-mouse CD16/32 Antibody | Biolegend | Cat#101332 |
| BV605 Rat Anti-Mouse CD71 | BD | Cat#563013 |
| APC anti-mouse Ly-6A/E (Sca-1) Antibody | Biolegend | Cat# 108112 |
| Brilliant Violet 421™ anti-mouse CD41 Antibody | Biolegend | Cat#133912 |
| Brilliant Violet 510 anti-mouse CD45 Antibody | Biolegend | Cat#103138 |
| V450 Rat Anti-Mouse Ly-6G | BD | Cat#560603 |
| Alexa Fluor® 647 Rat Anti-Mouse F4/80 | BD | Cat#565853 |
| APC anti-mouse CD3epsilon Antibody | Biolegend | Cat#100312 |
| PerCP/Cyanine5.5 anti-mouse CD19 Antibody | Biolegend | Cat#152406 |
| 7AAD | BD | Cat#559925 |
| PE Mouse Anti-Human CD34 | BD | Cat#555822 |
| APC Mouse Anti-Human CD235a | BD | Cat#551336 |
| FITC Mouse Anti-Human CD36 | BD | Cat#555454 |
| CD123 Monoclonal Antibody (6H6), PE-Cyanine7 | eBioscience | Cat#25-1239-42 |
| CD49d Antibody, anti-human, PE | Miltenyi Biotec | Cat#130-124-229 |
| FITC-Band3 | From our lab |  |
| BV421 Mouse Anti-Human CD34 | BD | Cat#1562577 |
| FITC anti-human CD38 Antibody | Biolegend | Cat#303503 |
| PE anti-human CD45RA Antibody | Biolegend | Cat#304107 |
| APC anti-human CD90 (Thy1) Antibody | Biolegend | Cat#328113 |
| Chemicals, peptides, and recombinant proteins | | |
| MethoCultTM M3434 | STEMCELL Technologies | Cat#03434 |
| MethoCultTM M3334 medium | STEMCELL Technologies | Cat#03334 |
| MS columns | Miltenyi Biotec | Cat# 130042401 |
| BCA protein assay kit | Pplygen | Cat# P1511 |
| anti-Biotin microbeads | Miltenyi Biotec | Cat# 130-109-485 |
| enhanced chemiluminescence | Sigma–Aldrich | Cat# WBLUM0100 |
| Click-iT™ Plus OPP Alexa Fluor™ 488 | Thermo Fisher | Cat# C10456 |
| Oligonucleotides | | |
| *CTNNB1-Forward Primer* | GAGCCTGCCATCTGTGCTCT |  |
| *CTNNB1-Reverse Primer* | ACGCAAAGGTGCATGATTTG |  |
| *MYC**-Forward Primer* | GTCAAGAGGCGAACACACAAC |  |
| *MYC**-Reverse Primer* | TTGGACGGACAGGATGTATGC |  |
| *CDK2*-Forward Primer | TTCTGCCATTCTCATCGG |  |
| *CDK2*-Reverse Primer | ATGGGTGTAAGTACGAACAGG |  |
| *CDK4-* Forward Primer | TCCCGAAGTTCTTCTGCAGT |  |
| *CDK4-Reverse* Primer | GTCGGCTTCAGAGTTTCCAC |  |
| *CDK6**-*Forward Primer | CGAACAGACAGAGAAACCAAAC |  |
| *CDK6-*Reverse Primer | AAGAAAGTCCAGACCTCGGA |  |
| *CDKN1B-*Forward Primer | GGCCTCAGAAGACGTCAAAC |  |
| *CDKN1B-*Reverse Primer | ACAGGATGTCCATTCCATGA |  |
| *CDKN1A**-*Forward Primer | CTGGAGACTCTCTGCAGGGTCGAAA |  |
| *CDKN1A-*Reverse Primer | GATTAGGGCTTCCTCTTGGAGAA |  |
| *CCND1-*Forward Primer | CGTGGCCTCTAAGATGAAGG |  |
| *CCND1-*Reverse Primer | CTGGCATTTTGGAGAGGAAG |  |
| *CCNE1-*Forward Primer | CTGGATGTTGACTGCCTTGA |  |
| *CCNE1-*Reverse Primer | TCCCCGTCTCCCTTATAACC |  |
| *shLuci*–Forward | CCGGCTCTTCTTTGAGTTCTACTCGAGTAGAACTCAAAGAAGAGTTTTTG |  |
| *shLuci*–Reverse | AATTCAAAAACTCTTCTTTGAGTTCTACTCGAGTAGAACTCAAAGAAGAG |  |
| *shGDF15#1*–Forward | CCGGCCGGATACTCACGCCAGAAGTCTCGAGACTTCTGGCGTGAGTATCCGGTTTTTG |  |
| *shGDF15#1*–Reverse | AATTCAAAAACCGGATACTCACGCCAGAAGTCTCGAGACTTCTGGCGTGAGTATCCGG |  |

**Mice cell staining system**

LT-HSC: Lin^-^Sca-1^+^c-Kit^+^CD34^-^CD135^-^

ST-HSC: Lin^-^Sca-1^+^c-Kit^+^CD34^+^CD135^-^

MPP: Lin^-^Sca-1^+^c-Kit^+^CD34^med^CD135^-^

CMP: Lin^-^Sca-1^-^c-Kit^+^CD34^+^CD16/32^low^

GMP: Lin^-^Sca-1^-^c-Kit^+^CD34^med^CD16/32^+^

MEP: Lin^-^Sca-1^-^c-Kit^+^CD34^low^CD16/32^low^CD150^+^CD105^low^

BFU-E: Lin^-^CD16/32^-^CD34^-^CD71^−/low^c-kit^+^

CFU-E: Lin^-^CD16/32^-^CD34^-^CD71^+^c-kit^+^

Erythroblast: Ter119^+^CD11b^-^CD45^-^Ly6C/Ly6G^-^CD44^+^

Reticulocyte: Ter119^+^CD11b^-^CD45^-^Ly6C/Ly6G^-^CD44^low^

*Flow Cytometric Detection of Murine Hematopoietic Stem and Progenitor Cells*

Mouse BM cells were harvested and centrifuged at 300 g for 10 minutes. The cells were mixed with the antibody mixture prepared according to Table S1, then incubated at 4℃ with shaking at 150 rpm in the dark for 30 minutes. Cells were washed by Buffer. 3×10⁶ cells were transferred to EP tubes, resuspended in antibody mixture as shown in Tables S2 and S3, and stained for LSK⁺ and LSK⁻ cells respectively. Incubation was performed at 22℃ in the dark for 30 minutes. Cells were washed once and resuspended in Buffer solution and transferred to flow cytometry tubes. 2 μL of 7AAD was added, and the cells were analyzed using a flow cytometer. The analysis results were processed with FlowJo software.

*Flow Cytometric Detection of Murine Erythroid Progenitor Cells*

Cells were stained with Table S1 according to the previous described, then resuspended in anti-Biotin Microbeads Buffer solution (1:4). The cells were placed on a horizontal shaker at 150 rpm in a 4℃ and incubated in the dark for 15 minutes. After washing, the cells were resuspended in 500 μL of Buffer solution, and added to a pre-rinsed MS column. The Lineage⁻ cells flowing out of the magnetic column were collected. After the complete outflow of the cell suspension, 500 μL of Buffer solution was added to the column for washing, and this operation was repeated twice. Subsequently, the cells were taken and incubated with antibodies listed in S4 at 22℃ in the dark for 30 minutes. 1 mL of Buffer was added, and the cells were centrifuged for 10 minutes to remove unbound antibodies. The cells were resuspended in Buffer solution and transferred to flow cytometry tubes. 2 μL of 7AAD was added, and the cells were analyzed using a flow cytometer. The analysis results were processed with FlowJo software.

*Flow Cytometric Detection of Murine Erythroid Cells, Granulocytes, Macrophages, T and B cells*

Murine BM cells were harvested and mixed with the antibodies prepared according to Table S5-S8. The cells were incubated on ice in the dark for 30 minutes. After the antibody incubation, wash away unbound antibodies in the supernatant. The cells were resuspended in Buffer solution and transferred to flow cytometry tubes. 2 μL of 7AAD was added (T and B cell were added DAPI), and the cells were analyzed using a flow cytometer. The analysis results were processed with FlowJo software.

**Table S1** Screening of erythroid stem progenitor cell staining system (1×10^7^cells/100 μL)

| Antibody |  | Volume (μL） |
| --- | --- | --- |
| Gr1 | Biotin | 0.25 |
| CD11b | Biotin | 0.25 |
| B220 | Biotin | 0.5 |
| CD3e | Biotin | 0.3 |
| Ter119 | Biotin | 1 |

**Table S2** LSK^+^ staining system (3×10^6^cells/100 μL)

| Antibody |  | Volume (μL） |
| --- | --- | --- |
| c-Kit | APC-Cy7 | 0.2 |
| CD34 | APC | 4 |
| Sca-1 | BV605 | 0.3 |
| CD135 | BV421 | 1.2 |
| Streptavidin | PercP | 1 |

**Table S3** LSK^-^ staining system (3×10^6^cells/100 μL)

| Antibody |  | Volume (μL） |
| --- | --- | --- |
| c-Kit | APC-Cy7 | 0.2 |
| CD34 | APC | 4.5 |
| Sca-1 | BV605 | 0.3 |
| CD16/32 | BV510 | 2.7 |
| Streptavidin | PercP | 1 |
| CD41 | Percp | 2.5 |
| CD105 | BV421 | 1.26 |
| CD150 | PE-Cy7 | 1.26 |

**Table S4** Erythroid progenitor cell staining system (2×10^5^ cells/50 μL)

| Antibody |  | Volume (μL） |
| --- | --- | --- |
| CD16/32 | BV421 | 1.2 |
| CD41 | BV421 | 1.2 |
| CD34 | APC | 1 |
| Sca-1 | APC | 0.2 |
| CD117 | APC-Cy7 | 0.2 |
| CD71 | BV750 | 0.4 |
| Streptavidin | PercP | 0.5 |

**Table S5** Erythroid cell staining system (1×10^6^ cells/50 μL)

| Antibody |  | Volume (μL） |
| --- | --- | --- |
| CD45 | APC-Cy7 | 0.25 |
| CD11b | APC-Cy7 | 0.25 |
| Ly6G/Ly6C | APC-Cy7 | 0.25 |
| Ter119 | BV421 | 0.3 |
| CD44 | APC | 0.5 |

**Table S6** Granulocyte staining system (4×10^6^ cells/130 μL)

| Antibody |  | Volume (μL） |
| --- | --- | --- |
| CD45 | BV510 | 0.65 |
| Gr1 | APC-Cy7 | 0.65 |

**Table S7** BM macrophage staining system (4×10^6^ cells/130 μL)

| Antibody |  | Volume (μL） |
| --- | --- | --- |
| Ly6G | V450 | 0.52 |
| Ter119 | BV421 | 0.78 |
| F4/80 | AF647 | 0.65 |

**Table S8** T cell and B cell staining system (4×10^6^ cells/130 μL)

| Antibody |  | Volume (μL） |
| --- | --- | --- |
| CD45 | APC-Cy7 | 0.65 |
| CD3e | APC | 1.3 |
| CD19 | PerCP-Cy5.5 | 3.25 |

**Human cell staining system**

HSC: CD34^+^CD38^low^CD90^+^CD45RA^-^

MPP: CD34^+^CD38^low^CD90^low^CD45RA^-^

CMP: CD34^+^CD38^+^CD123^+^CD45RA^-^

GMP: CD34^+^CD38^+^CD123^+^CD45RA^+^

MEP: CD34^+^CD38^+^CD123^-^CD45RA^-^

BFU-E: CD123^-^GPA^-^CD34^+^CD36^-^

CFU-E: CD123^-^GPA^-^CD34^-^CD36^+^

*Flow Cytometric Detection of Human Hematopoietic Stem and Progenitor Cells*

Cells were harvested and mixed with the antibodies prepared according to Table S9. The cells were incubated in the dark at RT for 30 minutes. After the antibody incubation, wash away unbound antibodies in the supernatant. The cells were resuspended in Buffer solution and transferred to flow cytometry tubes. 2 μL of 7AAD was added, and the cells were analyzed using a flow cytometer. The analysis results were processed with FlowJo software.

*Flow Cytometric Detection of Early and Terminal* *Erythroid Differentiation Cells*

Cells were harvested and mixed with the antibodies prepared according to Table S10-11. The cells were incubated in the dark at RT for 30 minutes. After the antibody incubation, wash away unbound antibodies in the supernatant. The cells were resuspended in Buffer solution and transferred to flow cytometry tubes. 2 μL of 7AAD was added, and the cells were analyzed using a flow cytometer. The analysis results were processed with FlowJo software.

**Table S9** HSPC differentiation staining system (1×10^5^cells/30 μL)

| Antibody |  | Volume (μL） |
| --- | --- | --- |
| CD38 | FITC | 1.25 |
| CD45RA | PE | 1.25 |
| CD90 | APC | 1.25 |
| CD34 | BV421 | 1.25 |
| CD123 | PE-Cy7 | 1.25 |

**Table S10** Early differentiation staining system (1×10^5^cells/25 μL)

| Antibody |  | Volume (μL） |
| --- | --- | --- |
| CD34 | PE | 2 |
| CD235a | APC | 1 |
| CD36 | FITC | 1 |
| CD123 | PE-Cy7 | 2 |

**Table S11** Terminal differentiation staining system (1×10^5^cells/25 μL)

| Antibody |  | Volume (μL） |
| --- | --- | --- |
| CD235a | APC | 1 |
| α4-integrin | PE | 0.5 |
| Band3 | FITC | 1.3 |

**Supplementary Figure Legends**

**
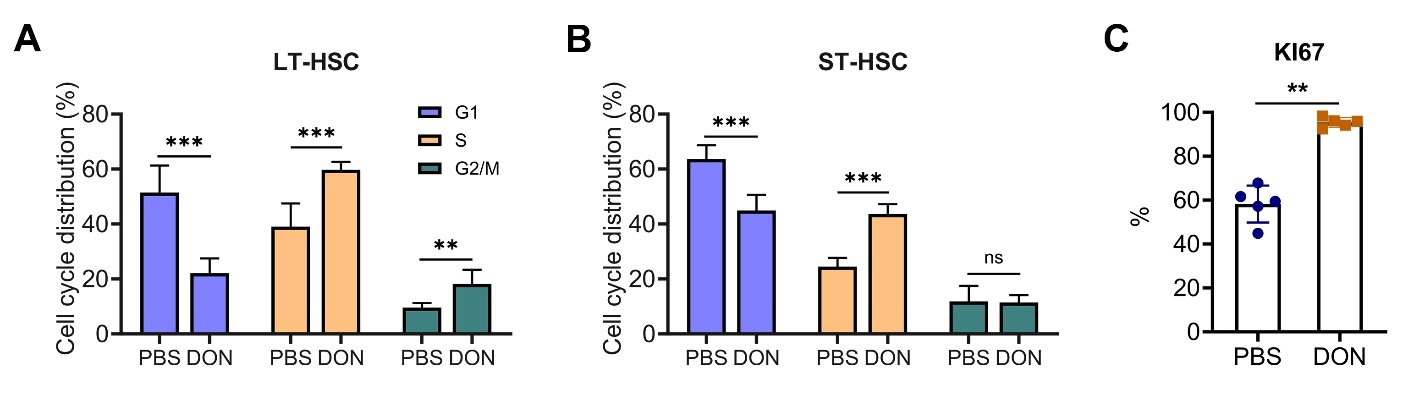
**

**Supplemental Figure 1.** **Deoxynivalenol (DON) activates the self‑renewal of HSCs.** Quantification analysis of cell cycle distribution of **A.** LT-HSC and **B.** ST-HSC in the bone marrow (BM) on day 1 following treatment with PBS or DON in mice; n=5–7 mice/group. **C.** Quantification analysis of Ki67^+^ cells of HSC in the mice BM on day 1 following treatment with PBS or DON; n=5 mice/group. Data are presented as mean ± SD. Comparisons between two groups were performing using Mann-Whitney test. Significance was set at ***p* < 0.01*,* and ****p* < 0.001.

**
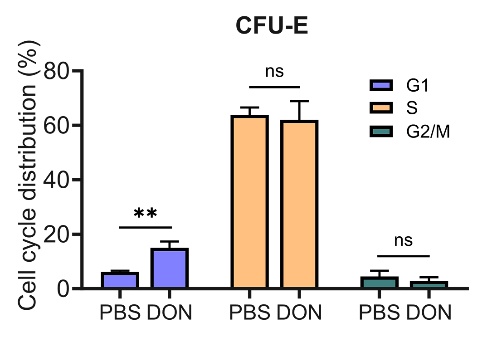
**

**Supplemental Figure 2.** Quantification analysis of cell cycle distribution of CFU-E in the BM on day 1 following treatment with PBS or DON in mice; n=5–7 mice/group. Data are presented as mean ± SD. Comparisons between two groups were performing using Mann-Whitney test. Significance was set at ***p* < 0.01.


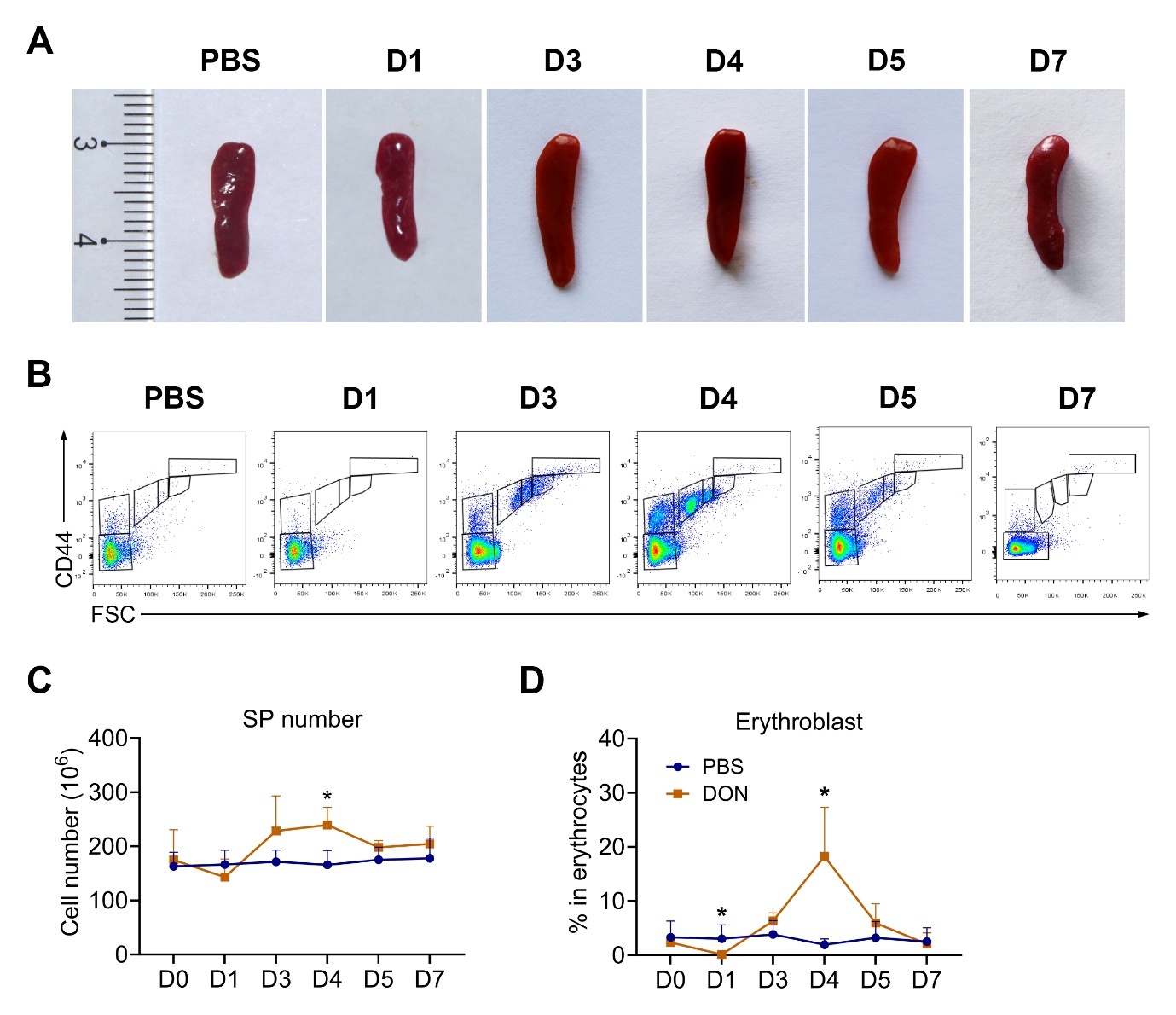


**Supplemental Figure 3. DON causes mild splenomegaly and increased erythropoiesis in mice.** **A.** Representative images of the change of mice spleen following treatment with PBS or DON. **B.** Representative plots of mice spleen erythropoiesis following treatment with PBS and DON. **C.** Trend of total spleen cells in PBS and DON-treated mice; n=3–5 mice/group. **D.** Percentage of erythroblast in spleen erythrocytes; n=3–5 mice/group. Data are presented as mean ± SD. Comparisons between two groups were performing using Mann-Whitney test. Significance was set at **p* < 0.05.

**
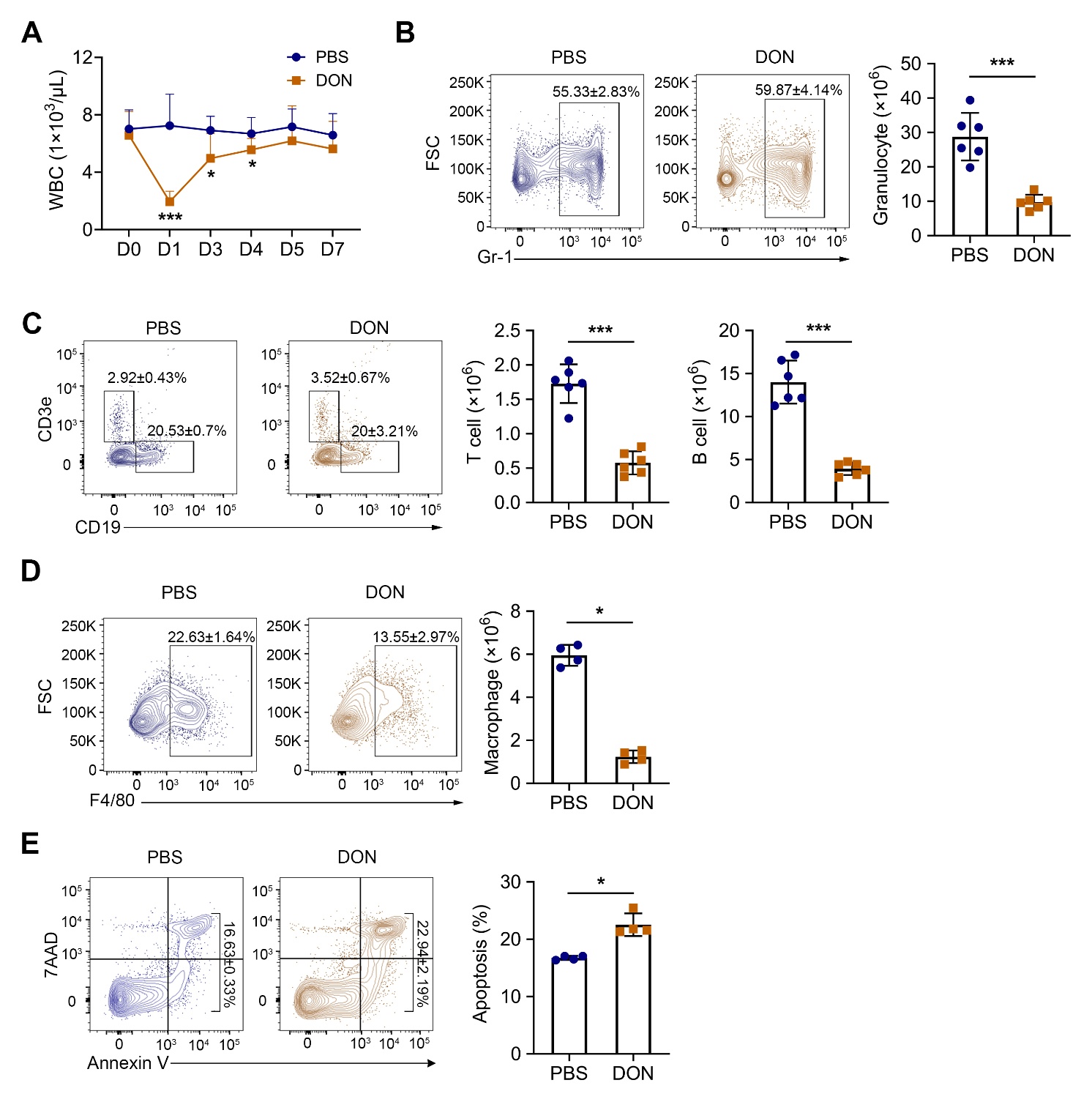
**

**Supplemental Figure 4.** **DON destroyed mice non-erythroid cells. A.** Complete blood count of DON treated mice. WBC, white blood cell. n=7 mice/group. **B.** Representative plot and quantification of the mice BM granulocytes on day 1 after mice treated with PBS or DON. n=6 mice/group. **C.** Representative plot and quantification of the mice BM T and B cells on day 1 following treatment with PBS or DON in mice. n=6 mice/group. **D.** Representative plot and quantification of the mice BM macrophages on day 1 following treatment with PBS or DON in mice. n=4 mice/group. **E.** Representative plot and quantification of the mice BM non-erythroid cells apoptosis treated with PBS or DON. n=4 mice/group. Data are presented as mean ± SD. Comparisons between two groups were performing using unpaired two-tailed Student’s *t*-test or Mann-Whitney test. Significance was set at**p* < 0.05*,* and ****p* < 0.001.


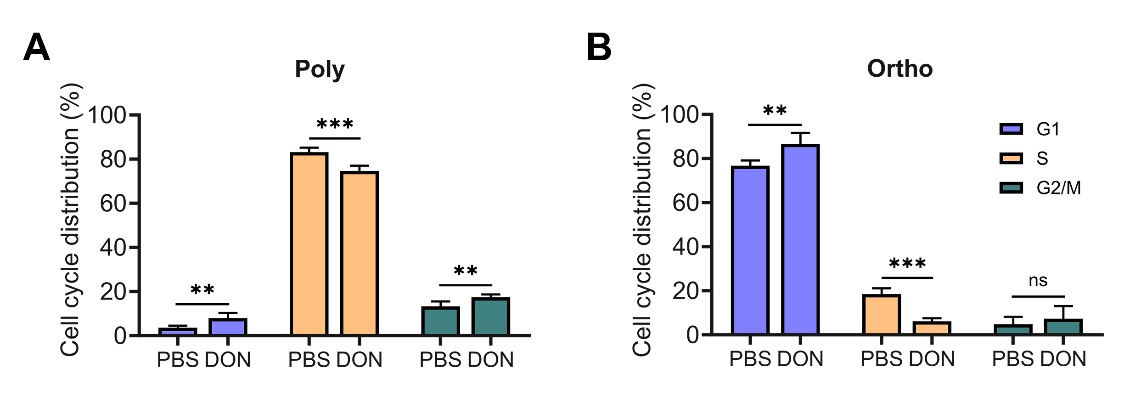


**Supplemental Figure 5. DON arrest G1/S phase of erythroblasts.** Quantification analysis of cell cycle distribution of **A.** polychromatic erythroblasts, and **B.** orthochromatic erythroblasts on day 1 following treatment with PBS or DON in mice; n=5–7 mice/group. Data are presented as mean ± SD. Comparisons between two groups were performing using Mann-Whitney test. Significance was set at ***p* < 0.01*,* and ****p* < 0.001.


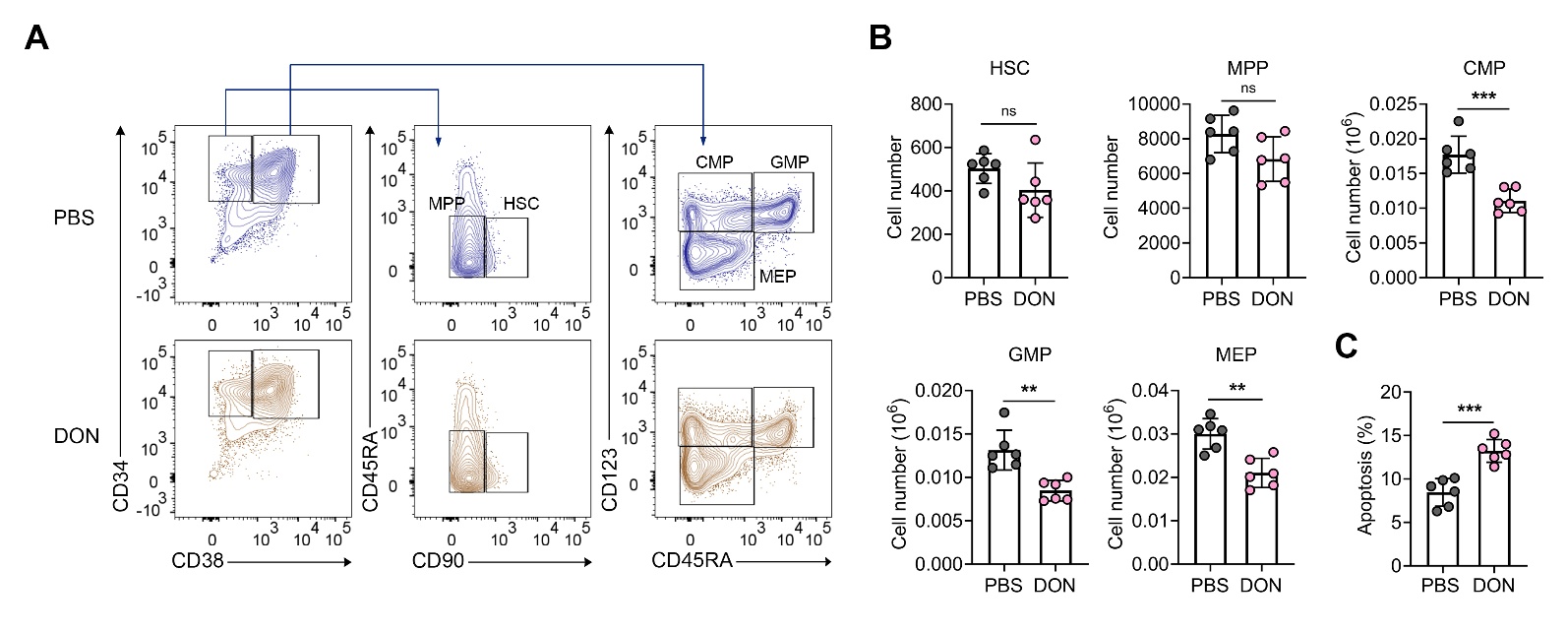


**Supplemental Figure 6. DON causes development imbalance in human** **hematopoietic progenitor cells.** **A.** Representative flow cytometry analyses plots of the human hematopoietic progenitor cells treated with PBS and 100 ng/mL DON on day 4. The number of **B.** HSC, MPP, CMP, GMP, and MEP on day 4 in the CD34^+^ cell culture; n=6. **C.** Quantification analysis of apoptotic human hematopoietic progenitor cells on day 4 in the presence of PBS and DON (0, and 100 ng/mL); n=6. Data are presented as mean ± SD. Comparisons between two groups were performing using unpaired two-tailed Student’s *t*-test. Significance was set at**p* < 0.05*,* and ****p* < 0.001.

**
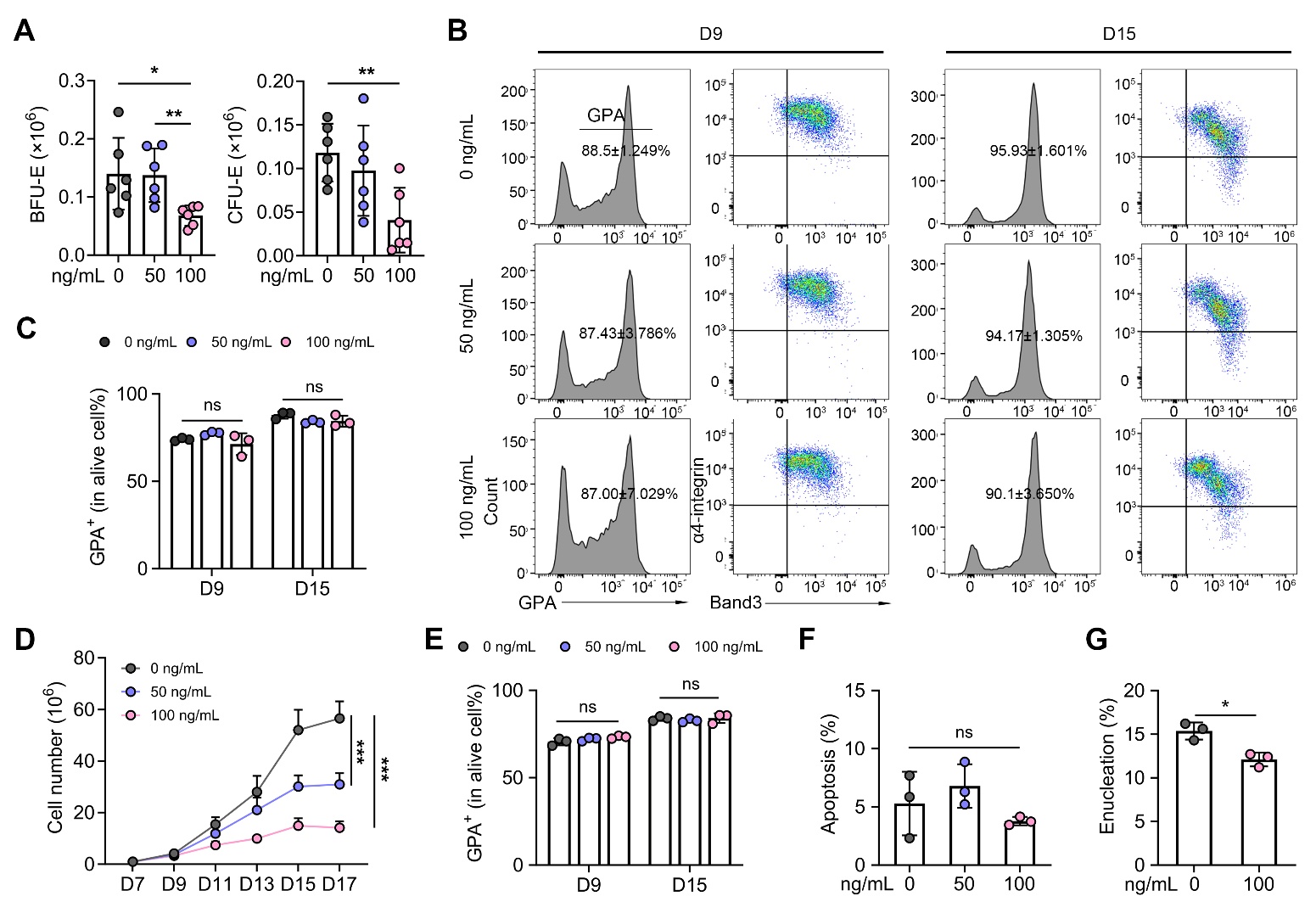
**

**Supplemental Figure 7.** **DON has little effect on human terminal erythroid differentiation.** **A.** The number of BFU-E and CFU-E cells on day 5 in the CD34^+^ cell culture; n=6. **B.** Representative flow cytometry analyses plot of terminal erythroid differentiation gating strategy treated with PBS and different concentration of DON. **C.** The percentage of GPA^+^ cells on day 9 and 15 of erythroid cells treated with DON (0, 50, 100 ng/mL) from day 2. n=3. **D.** Growth curves of erythroid cells treated with DON (0, 50, 100 ng/mL) from day 7. n=5. **E.** Rate of GPA^+^ cells on day 9 and 15 of CD34^+^ cell culture with DON treated from day 7. n=3. **F.** Rate of apoptosis cells in human erythroid cells with DON treated from day 7 on D11. n=3. **G.** Rate of enucleation on day 13 of CD34^+^ cell culture with DON treated from day 7. n=3. Data are presented as mean ± SD. ANOVA with Tukey's *post hoc* test was used to calculate statistical significance among multiple groups. Significance was set at **p* < 0.05*, **p* < 0.01*,* and ****p* < 0.001.


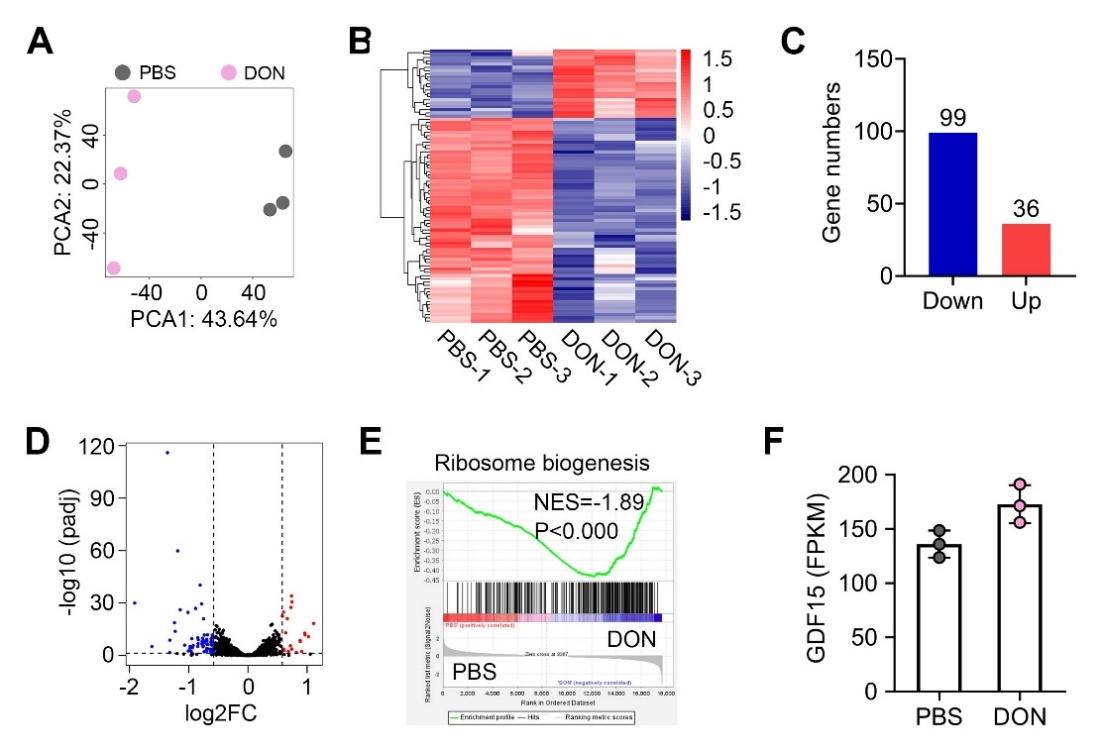


**Supplemental Figure 8.** **DON has little effect on transcriptional expression profile.** **A.** Principal component analysis (PCA) of all RNA-seq samples. The first and second components are shown. **B.** Heatmap of the differentially expressed genes. **C.** Number of differentially expressed genes in PBS-treated and DON-treated erythroid progenitor cells. **D.** Volcano plots showing transcriptomic changes in PBS-treated and DON-treated erythroid progenitor cells. **E.** GSEA performed on transcriptomic data shown to reveal ribosome biogenesis, NES, normalized enrichment score. **F.** The FPKM of GDF15 in PBS and DON-treated erythroid progenitor cells.


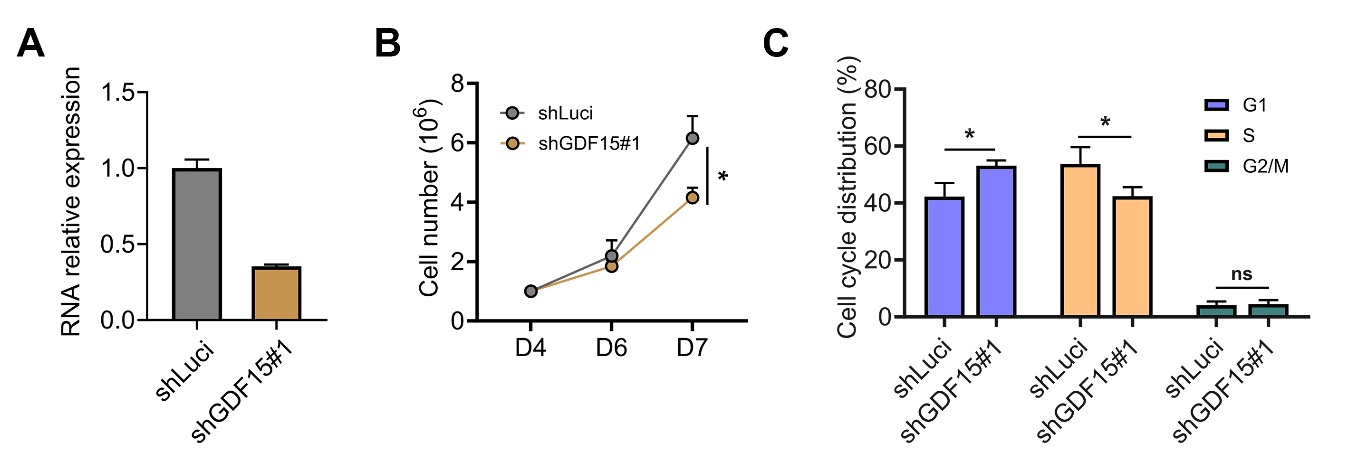


**Supplemental Figure 9.** **Loss of GDF15 impairs human erythroid proliferation and cell cycle. A.** The qRT-PCR results showing GDF15 mRNA expression levels in control and GDF15-knockdown cells. n=3. **B.** Growth curves of cells transduced with lentivirus containing shLuci or shGDF15 in erythroblasts cultured for 4, 6, and 7 days; n=4. **C.** Quantification of cell cycle distribution; n=4. Data are presented as mean ± SD. Comparisons between two groups were performing using Mann-Whitney test. Significance was set at **p* < 0.05.
